# Insight into the potential role of ferroptosis in neurodegenerative diseases

**DOI:** 10.3389/fncel.2022.1005182

**Published:** 2022-10-27

**Authors:** Yingying Ji, Kai Zheng, Shiming Li, Caili Ren, Ying Shen, Lin Tian, Haohao Zhu, Zhenhe Zhou, Ying Jiang

**Affiliations:** ^1^The Affiliated Wuxi Mental Health Center of Jiangnan University, Wuxi Central Rehabilitation Hospital, Wuxi, China; ^2^Rehabilitation Medicine Center, The First Affiliated Hospital of Nanjing Medical University, Nanjing, China

**Keywords:** ferroptosis, iron, Alzheimer's disease, neurodegenerative diseases, treatment

## Abstract

Ferroptosis is a newly discovered way of programmed cell death, mainly caused by the accumulation of iron-dependent lipid peroxides in cells, which is morphologically, biochemically and genetically different from the previously reported apoptosis, necrosis and autophagy. Studies have found that ferroptosis plays a key role in the occurrence and development of neurodegenerative diseases, such as Alzheimer's disease, Parkinson's disease and vascular dementia, which suggest that ferroptosis may be involved in regulating the progression of neurodegenerative diseases. At present, on the underlying mechanism of ferroptosis in neurodegenerative diseases is still unclear, and relevant research is urgently needed to clarify the regulatory mechanism and provide the possibility for the development of agents targeting ferroptosis. This review focused on the regulatory mechanism of ferroptosis and its various effects in neurodegenerative diseases, in order to provide reference for the research on ferroptosis in neurodegenerative diseases.

## Introduction

Iron is involved in oxygen transport and cellular respiration, DNA synthesis and cell division, cellular metabolism and neurotransmission, which are essential for maintaining the body's function and daily metabolism. The ability of iron to circulate in an oxidative state in the body is fundamental to its biological function, and excess iron can lead to oxidative stress damage to biomolecules, as well as cellular dysfunction. However, with the increase of age, the accumulated iron in the brain will increase the risks of neurodegenerative diseases (Belaidi and Bush, 2016; Eid et al., 2017).

**Figure 1:**
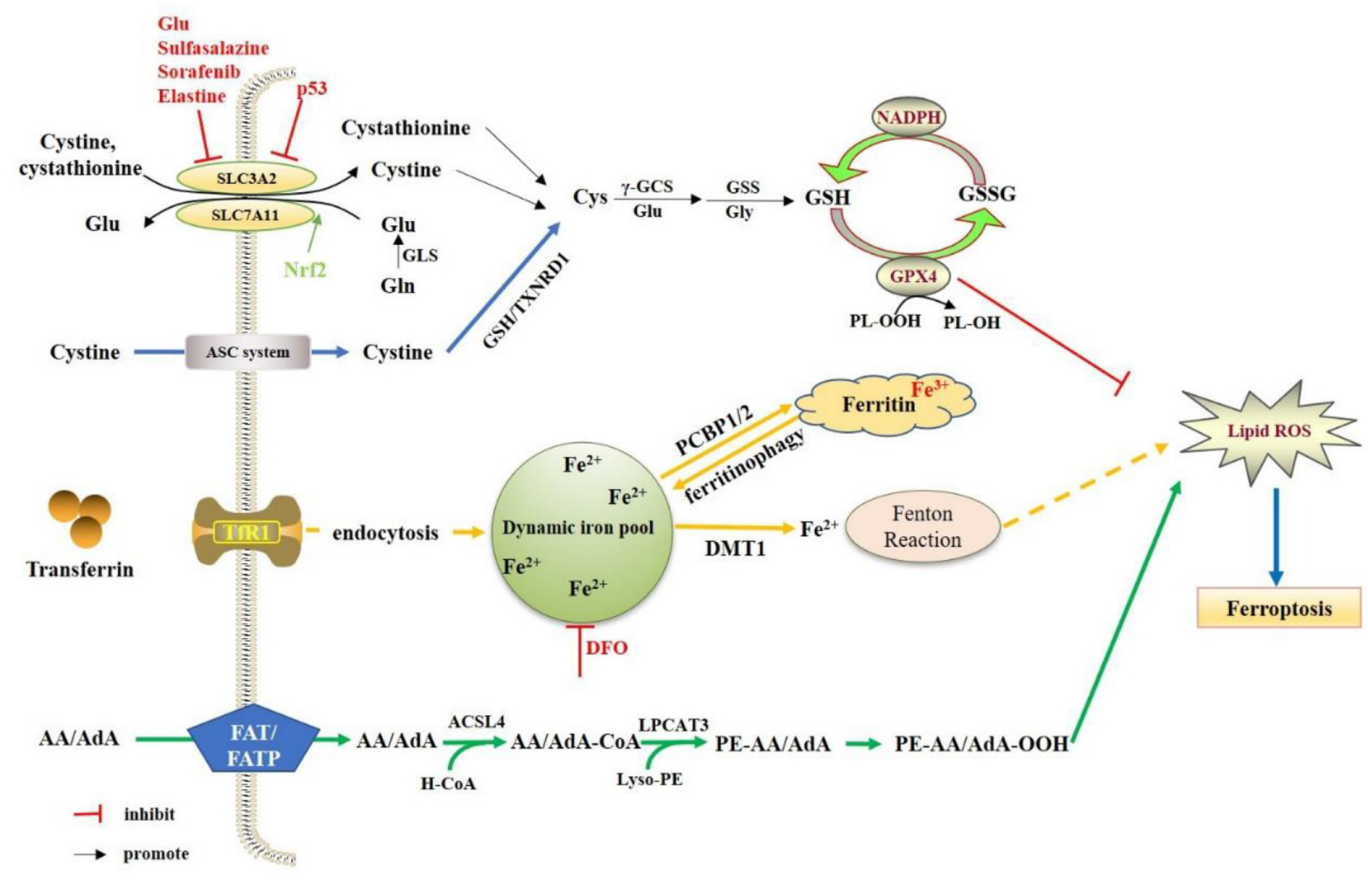
Regulation mechanisms of ferroptosis. The System Xc- is a heterodimeric amino acid transporter composed of two subunits (SLC7A11and SLC3A2). Under oxidative conditions, System Xc-extracellular uptake of cystine to release glutamate. The cellular cystine will be reduced to cysteine. Cysteine is gradually produced by γ-GCS and GSS. The GSH molecules of GPX4 act as electron donor to reduce phospholipid hydrogen peroxide (PL-OOH) to the corresponding alcohol. NADPH can also reduce GSSG (oxidized GSH) to GSH by glutathione reductase. The extracellular iron will be mediated by transferrin and TfR1 into the cell. Then Fe3+ is reduced to Fe2+, and Fe2+ will be transported to the dynamics iron pool, as well as stored in ferritin. In the presence of Fe2+, hydrogen peroxide can generate hydroxyl radicals (OH–) through the fenton reaction, thereby promoting the oxidation of AA/AdA on the cell membrane. ACSL4 incorporates AA/AdA into the membrane as AA/AdA-CoA, and LPCAT3 inserts the acyl group into the lysophospholipid as PE-AA/AdA, of which will lead to the accumulation of PE- AA/AdA-OOH.

Ferroptosis is an iron-dependent, novel cell death mode, which is significantly different from apoptosis, cell necrosis and autophagy. The main mechanism is that under the action of ferrous iron or lipoxygenase, iron catalyzes liposomal peroxidation of highly expressed unsaturated fatty acids on cell membranes, thereby inducing cell death (Dixon et al., 2012; Nikseresht et al., 2019). The morphological features of ferroptosis are mitochondrial atrophy, increased bilayer membrane density, and loss of mitochondrial inner membrane cristae, with the intact cell membrane remaining and the normal size of the nucleus, as well as no chromatin condensation (Alborzinia et al., 2018; Ou et al., 2022). Numerous studies have shown that ferroptosis is also related to a reduction in the expression of glutathione and glutathione peroxidase 4 (GPX4) in the antioxidant system of cells (Zhan et al., 2022; Zhao et al., 2022; Zhu et al., 2022). Lipid peroxides cannot be metabolized by the reduction reaction catalyzed by GPX4, and lipids are oxidized by ferrous iron in Fenton reaction to generate a large amount of reactive oxygen species to promote ferroptosis (Alborzinia et al., 2018; Torii, 2018; He et al., 2020). Therefore, the essence of ferroptosis is the metabolic disorder of intracellular lipid oxides, which are then abnormally metabolized under the catalysis of iron ions to produce a large amount of lipids to destroy the intracellular redox balance and attack biological macromolecules, triggering programmed cell death (Figure 1).

In recent years, studies have found that ferroptosis plays an extremely important role in neurodegenerative diseases with a common regulatory mechanism. This review will focus on ferroptosis and neurodegenerative diseases, such as Alzheimer's disease (AD), vascular dementia (VD), Parkinson's disease (PD), Huntington's disease (HD), amyotrophic lateral sclerosis (ALS) and traumatic brain injury (TBI), as well as the potential therapeutic effects of targeting ferroptosis (Table 1).

**Table 1:** Targeting ferroptosis for the potential treatment of neurodegenerative diseases.

**Diseases**	**Types**	**Name**	**Potential mechanism**
AD	Iron chelators	DFO DFP	Improve the excretion of iron, reduce the content of iron in the body and its pathological deposition in various organs
	Quinoline derivatives	Clioquinol	Downregulate the expression of ß and y secretases and APP in the brain, degrade oligomeric tau and reduce tau tangles
	Antioxidants	Vitamin E	Reduce lipid peroxidation and attenuate iron morphology
		a-LA	Block iron overload, lipid peroxidation and inflammation
		Se-containing compounds	Upregulate GPX4 to inhibit iron toxicity
		Fer-1	Inhibit ROS levels and downregulate Nrf2 and GPX4
	Hepcidin	Hepcidin	Inhibit FPN1, TfR1 and DMT1 to reduce neuronal iron uptake and release
	GBH	GBH	Inhibit lipid peroxidation and restore the expression of ferritin GPX4, HO-1, COX-2 altered by RSL3
VD	Antioxidants	Fer-1	Inhibit ROS levels and downregulate Nrf2 and GPX4
	Thiazolidinedione	ROSI	Inhibits ACSL4 activity, reduce lipid peroxidation and oxidative stress
PD	Iron chelator	DFP	Reduce oxidative stress and increase dopamine activity
	Quinoline derivatives	Clioquinol	Chelate iron and antioxidant
HD	Iron chelator	DFO	Improve the excretion of iron, reduce the content of iron in the body and its pathological deposition in various organs
	Antioxidant	Fer-1	Inhibit oxidative lipid damage
TBI	Iron chelators	DFO	Reduce the iron overload, and relieve acute oxidative brain injury
		HBED	
	Ferroptosis inhibitor	Liproxstatin-1	Reduce cerebral edema and blood-brain barrier permeability

## Mechanisms of ferroptosis

### Transport and storage of iron in the brain

The neuronal iron metabolism related protein, TfR1 (transferrin receptor protein 1), is highly expressed on the surface of neurons (Giometto et al., 1990). Similar to BMECs, iron enters neurons through clathrin-mediated phagocytosis of holo-Tf/TfR1, and exits endosomes in the form of reduced Fe2+ through DMT1 (Burdo et al., 2001). NTBI (Non-transferrin-bound iron) can also enter neurons independently of Tf in a DMT1 (Divalent metal transporter 1)-dependent manner. Prion protein (PrPC), as the ferroreductase partner of DMT1, mediates the uptake of PrPc/DMT1 in the plasma membrane in the form of a complex of iron ions (Shih et al., 2003; Tripathi et al., 2015). Brain iron deficiency and increased holo-Tf uptake can be found in PrPC knockout mice (Singh, 2014). In the brain, divalent iron ions are normally metabolized in the neuronal cytoplasm, and stored in ferritin in the form of trivalent iron ions. When neurons are iron deficient, ferritin can be degraded by lysosomes, releasing stored iron to meet the normal physiological needs of neurons (Connor et al., 1992; Mancias et al., 2014; Raha et al., 2022). Studies have shown that ferritin in the brain increases with age (Belaidi et al., 2018), which is positively correlated with cognitive dysfunction. The etiology of cognitive dysfunction in the elderly is closely related to the iron overload. Iron homeostasis can also be regulated at the translation level. Iron regulatory protein 2 (IRP2), an RNA binding protein, controls the translation of a group of mRNAs involved in iron homeostasis. In the untranslated regions (UTRs) of genes encoding a variety of iron regulatory molecules (including DMT1 and TFR1), the IRP1 and IRP2 bind to the iron response elements (IREs). In an iron-deficient state, the combination of IRP2 and IREs can maximize intracellular iron levels. When the iron content increases, the extracellular iron regulatory pathway (IRE/IRP system) will be activated to weaken the iron overload state (Rouault, 2006; Sanchez et al., 2007; Hentze et al., 2010). In addition, nuclear receptor coactivator 4 (NCOA4) can degrade ferritin to mediate iron autophagy, and release free iron in the process, which can also lead to an increase in intracellular Fe2+ and ferroptosis (Li W. Y. et al., 2021). Iron-responsive element-binding protein 2 (IREB2) is a regulator of iron metabolism, which can up-regulate the expression of ferritin light chain and ferritin heavy chain in the cytoplasm during iron metabolism, and alleviate erastin-induced ferroptosis (Mishima, 2022). Nuclear factor E2-related factor 2 (Nrf2) can reduce the expression of TfR1, regulate iron metabolism, maintain the balance of intracellular iron homeostasis, and limit the production of reactive oxygen species (ROS), thereby reducing ferroptosis (Li S. W. et al., 2021).

### The role of the glutamate/cysteine antiporter in ferroptosis

Cystine uptake by the glutamate/cysteine antiporter (System Xc-), including a 12-pass transmembrane protein transporter solute carrier family 7 member 11 (SLC7A11) and a single-channel transmembrane regulatory protein solute carrier family 3 member 2 (SLC3A2), is inhibited in ferroptosis (Dixon et al., 2012). Thus, inhibition of the System Xc- results in the depletion of intracellular cysteine (Ma et al., 2021). Cysteine plays an important role in the biosynthesis of glutathione (GSH). GSH, as a substrate of GPX4, is required for its lipid repair function. GSH depletion through cysteine starvation results in the loss of GPX4 activity, as well as the accumulation of unrepaired lipid peroxides and iron toxicity (Angeli et al., 2014). GPX4 converts reduced glutathione to oxidized glutathione (GSSG) to reduce lipid hydrogen peroxide to the corresponding alcohol or free hydrogen peroxide to water (Gaschler et al., 2018). Selenium (Se) is a key regulator of GPX4 activity. Wild-type GPX4 containing Se can effectively reduce peroxides to the corresponding alcohols, thereby preventing ferroptosis (Ingold et al., 2018). GSH is also a natural ligand for Fe2+ in the labile iron pool (LIP), which is an exchange pool for loosely ligated iron in neurons (Hider and Kong, 2011), and glutathione binds Fe2+ in LIP to prevent iron oxidation, which not onlymaintains Fe2+ solubility but also prevents Fe2+ from acting as a catalyst to generate a potent oxidant, hydroxyl radical, from physiologically available hydrogen. Therefore, direct inhibition of GSH synthesis triggers ferroptosis.

### The role of lipid peroxidation in ferroptosis

Lipid metabolism is also closely related to ferroptosis. Nitrogen oxides (NOXs) provide a source of accumulation of ROS in erastin-induced iron sickness, and it has been reported to modulate the sensitivity of tumor cells to erastin (Dixon et al., 2012). On the other hand, the production of membrane lipid peroxidation is also a source of ROS, which drives iron toxicity. The abundance and location of polyunsaturated fatty acids (PUFAs) determine the degree of lipid peroxidation that occurs in cells, and lead to ferroptosis. The most susceptible lipids are phospholipids containing polyunsaturated fatty acids (PUFA-PLs), which can lead to subsequent cell death (Doll et al., 2017). Free PUFAs need to be esterified to form membrane phospholipids and oxidized to iron ion signals to synthesize lipid signals, especially phospholipids containing phosphatidylethanolamine (PE) and arachidonic acid or epinephrine moieties (Kagan et al., 2017). In the membrane lipid environment, PUFAs are specifically peroxidized in iron toxicity (Doll et al., 2017; Kagan et al., 2017). There are three main classes of lipid oxidases: cyclooxygenases (cox), cytochrome p450s (CYPs), and lipid oxidases (LOXs), of which LOX enzymes have been found to be most important for ferroptosis. LOXs are a class of non-heme iron-containing enzymes that catalyze the deoxygenation of PUFAs (Shintoku et al., 2017).

## Relationship between ferroptosis and neurodegenerative diseases

### AD

In addition to β-amylase deposition and accumulation of intracellular neurofibrillary tangles (NFTs) composed of tau proteins, abnormal deposition of iron in the brain is a common feature of AD. The effects of iron on AD have been attributed to its interaction with AD pathological central proteins (amyloid precursor protein and tau protein) and/or through iron-mediated generation of prooxidative molecules such as hydroxyl radicals. However, the source of iron accumulation in brain pathologically relevant regions and its contribution to AD remain unclear. The potential reason for iron accumulation is that senescent cells within tissues increase with age, and these cells trigger inflammation and contribute to various pathologies associated with aging. The accumulation of iron makes aging tissues susceptible to oxidative stress, leading to cellular dysfunction and ferroptosis. In addition, elevated brain iron levels are associated with AD progression and cognitive decline. Elevated brain iron levels, a hallmark of AD, can be pharmacologically modulated to mitigate the effects of age-related dysregulation of iron balance and improve disease outcomes (Masaldan et al., 2019a).

A meta-analysis involving 300 AD cases in 19 studies reported that iron levels were significantly elevated in multiple regions of the cerebral cortex, although iron levels varied across regions and studies (Tao et al., 2014). Iron accumulation may be detrimental, as elevation of iron itself may lead to neurodegeneration (Schneider et al., 2012), possibly by inducing oxidative stress and ferroptosis (Stockwell et al., 2017). High brain iron levels, cerebrospinal fluid ferritin (Ayton et al., 2015, 2017a), and quantitative susceptibility maps have been found to have the potential to predict AD clinical severity and cognitive decline (Ayton et al., 2017b). The relationship between postmortem brain iron levels and AD clinical and pathological diagnosis, severity, and rate of cognitive decline in the 12 years preceding death was also investigated in 209 AD patients. It was found that the iron content in the brains of AD patients was significantly increased, and it was significantly related to cognitive function. Therefore, cortical iron may contribute to the deterioration of cognitive function in AD underlying proteinopathies by inducing oxidative stress or ferroptosis, or by being associated with inflammatory responses (Ayton et al., 2020). Another study found that iron deposition in the frontal lobe, parietal lobe, temporal lobe, caudate nucleus, putamen, globus pallidus, cingulate cortex, amygdala, and hippocampus of AD patients was higher than that of healthy controls (Tao et al., 2014), and histological differences in the intensity of iron accumulation in the frontal cortex of AD subtypes can be used not only to distinguish sporadic (late-onset) from familial (early-onset) (Bulk et al., 2018a), but also to correlate with disease severity (van Duijn et al., 2017; Bulk et al., 2018b).

The accumulation of iron has been proven to accelerate the deposition of senile plaques and the generation of neurofibrillary tangles (Becerril-Ortega et al., 2014; Kim et al., 2018). Autopsy evidence and MRI analysis provide evidence that there was substantial iron deposition not only in senile plaques (James et al., 2017), but also at sites of cortical tau protein accumulation (Spotorno et al., 2020), suggesting a potential interaction of iron with senile plaques and neurofibrillary tangles. Perturbation of iron homeostasis is one of the key factors in Aβ deposition. High intracellular iron concentration enhances the IRP/IRE interaction and induces upregulation of APP. The enzymes that cleave APP are called α- and β-secretases, which are tightly balanced and regulated by furin (Silvestri and Camaschella, 2008; Guillemot et al., 2013). In the presence of iron excess, more β-secretase is activated when α-secretase is inhibited by furin injury (Silvestri and Camaschella, 2008). Up-regulated APP is cleaved by more β-secretase Aβ40/42, accelerating Aβ deposition. At the same time, the application can no longer assist FPN1, resulting in impaired iron efflux and aggravated iron deposition (Ward et al., 2014). It was suggested that in the absence of redox metals, Aß is nontoxic, and the aggregation of Aß requires the participation of metals (Li et al., 2013; Belaidi and Bush, 2016). Soluble Aß binds to Fe3+ when extracellular iron increases, removing excess iron, but it is difficult to separate after interaction. Aß can promote the reduction of Fe3+ to Fe2+, and ROS released in the process make Aß easily and rapidly to deposit and form more senile plaques (Ha et al., 2007). The interaction of iron with APP and Aß greatly increased the rate and extent of senile plaque formation (Rottkamp et al., 2001). Therefore, iron deposition could be included in the “Aβ cascade hypothesis” of AD (Peters et al., 2018). Iron can also interact with tau protein. Decreased soluble tau protein in the brains of AD patients increases cerebral iron deposition by inhibiting FPN1 activity (Lei et al., 2012). Conversely, high-iron diet led to cognitive decline in mice, abnormally increased neuronal tau phosphorylation, and abnormal expression of insulin pathway-related proteins. Insulin supplementation reduces iron-induced tau phosphorylation (Wan et al., 2019), suggesting that iron deposition may lead to tau hyperphosphorylation by interfering with insulin signaling. *In vivo* studies have found that iron can participate in tau hyperphosphorylation by activating the cyclin-dependent kinase 5 (CDK5)/P25 complex and glycogen synthase kinase 3β (GSK-3β) (Guo et al., 2013). Excessive intracellular Fe2+-induced generation of oxygen free radicals can also promote tau hyperphosphorylation by activating extracellular signal-regulated kinase 1/2 (Erk1/2) or mitogen-activated protein kinase (MAPK) signaling pathways (Chan and Shea, 2006; Munoz et al., 2006).

Glial activation and neuroinflammation have been shown to be prominent features of AD pathology (Newcombe et al., 2018; Leng and Edison, 2021). Microglia are highly responsive cells that respond to increased iron levels in the brain. When iron levels are elevated in the brain, microglia are activated (Meng et al., 2017) with increased volume and decreased length (Rathnasamy et al., 2013; Donley et al., 2021). Iron may activate microglia *via* nuclear factor-kb (NF-KB)-mediated pro-inflammatory cytokines (Meng et al., 2017). Upon activation, more ferritin will be expressed to remove extracellular iron (Streit et al., 2022), leading to intracellular iron retention (Kenkhuis et al., 2021), increased TNFα expression (Holland et al., 2018), and eventual infiltration as a ß-plaque (Peters et al., 2018; Kenkhuis et al., 2021). It can also interact with APP to promote the formation of Aβ (Tsatsanis et al., 2021). Conversely, in an environment with elevated iron levels, Aß formation leads to increased IL-1ß expression in microglia, exacerbating pro-inflammatory effects (Nnah et al., 2020). Astrocytes are highly resistant to metal-induced toxicity in the brain (Kress et al., 2002), serving as a key cell type for maintaining the homeostasis of the extracellular environment and supporting normal neuronal function (Abbott et al., 2006). Under the high-iron environment, the levels of glutathione, catalase, and manganese superoxide dismutase are significantly elevated in astrocytes to counteract oxidative stress (Iwata-Ichikawa et al., 1999; Shih et al., 2003). However, astrocytes were found to be activated by increased glial fibrillary acidic protein (GFAP) (Kress et al., 2002). Activated astrocytes release inflammatory mediators, induce oxidative stress, and promote the formation of Aß and tau tangles, hindering Aß clearance (Dolotov et al., 2022).

Abnormal expression of GPX4 mRNA and its protein levels was found in AD patients and mouse brains (Yoo et al., 2012; da Rocha et al., 2018). In glial cells, mild hypoxia can reduce the level of GSH, which is used for GPX4 biosynthesis (Makarov et al., 2006). In a mouse model of AD, GSH expression was reduced in the cortex and positively correlated with cognitive decline (Karelson et al., 2001). GSH levels in the frontal lobe and hippocampus may serve as biomarkers for predicting AD and mild cognitive impairment (Karelson et al., 2001; Ayton et al., 2020). xCT activity determines GSH availability, and subsequent GPX4 activity in the brain (Ashraf et al., 2020). Furthermore, studies have found that most of the proteins involved in ferroptosis can be regulated by Nrf2 (Habib et al., 2015; Lane et al., 2021). The genes of interest included FPN1, GSH and SLC7A11 encoding xCT. The level of Nrf2 in the brain decreases with age, as well as in AD patients (Osama et al., 2020), so the brain of AD patients is more prone to ferroptosis (Habib et al., 2015). GPX4 expression has been reported to be reduced in both AD mouse models and AD patient brains (Ansari and Scheff, 2010; Yoo et al., 2010). GPX4 knockout mice were shown to have significant hippocampal neuronal loss and cognitive impairment (Yoo et al., 2010; Hambright et al., 2017). A diet deficient in vitamin E, an antioxidant with anti-ferroptosis activity, simultaneously results in hippocampal neurodegeneration and worsens behavioral dysfunction; the ferroptosis inhibitor liproxstatin 1 improves cognitive function and neurodegeneration in mice (Hambright et al., 2017). In addition to animal models, autopsy results of AD patients showed down-regulation of GPX4, up-regulation of arachidonic acid 12/15 lipoxygenase (ALOX15), and enhanced lipid peroxidation, and 4-hydroxynonenal (4-HNE) in AD patient brains was elevated (Yoo et al., 2010). 4-HNE has the potential to modify proteins involved in antioxidant and energy metabolism, promoting Aβ deposition and fibrogenesis (Seibt et al., 2019). These results indicate that ferroptosis plays a key role in AD, which can cause neuronal loss and cognitive decline. Therefore, modulating brain iron metabolism and reducing neuronal ferroptosis may be a promising approach for the treatment of AD.

Deferoxamine (DFO) and deferiprone (DFP) are commonly used clinical iron chelators. The clinical efficacy of DFO in the treatment of AD is as high as 50%. However, the side effects, such as weight loss and loss of appetite (Mclachlan et al., 1991), limit its clinical application. Besides, DFO is difficult to pass through the blood-brain barrier (BBB) (Ward et al., 1995; Ben Shachar et al., 2004), which could be solved by intranasal administration of DFO nanoparticles (Rassu et al., 2015). Compared to DFO, DFP could cross the BBB and is safer (Gallie and Olivieri, 2019). In a randomized controlled trial, DFP improved neurological scores and iron-related neurological symptoms (Abbruzzese et al., 2011; Klopstock et al., 2019).

Quinoline and its derivatives, which could chelate with iron, zinc, and copper, have the potential to improve cognition, reduce Aβ deposition, and promote Aβ degradation in AD animal models (Grossi et al., 2009; Crouch et al., 2011). Clioquinol could downregulate ß and y secretase and APP expression in the brain (Wang et al., 2012), as well as degrade oligomeric tau protein and reduce tau tangles (Lin et al., 2021). Besides, clioquinol has been proven to slow down cognitive decline in patients with severe AD and reduce Aß42 in cerebrospinal fluid (Ritchie et al., 2003). Studies have also found that vitamin E (an antioxidant) can reduce lipid peroxidation in the brain, reduce iron morphology on neurons, and improve cognitive function in GPX4 knockout mice. However, in a randomized clinical trial, vitamin E showed no benefit in patients with AD or mild cognitive impairment (Farina et al., 2017), while it could accelerate cognitive decline (Lloret et al., 2009). Therefore, the application of vitamin E in AD remains questionable, and more clinical trials are needed to determine its effect. Alpha-lipoic acid (a-LA) was also proven to improve cognitive impairment, slow cognitive decline (Fava et al., 2013), block iron overload, lipid peroxidation, and inflammatory responses in AD patients (Zhang et al., 2018). Se is present in various proteins in the body, such as GPX4, and has antioxidant activity. Se-containing compounds may inhibit iron toxicity by upregulating GPX4 and improve cognitive function in AD patients (Gwon et al., 2010; Cardoso et al., 2019). Ferrostatin-1 (Fer-1) is a common ferritin inhibitor and a free radical scavenger, which is much more effective than phenolic antioxidants (Miotto et al., 2020). Fer-1 could alleviate angiotensin II-induced astroglial inflammation and iron degeneration by inhibiting ROS levels and downregulating Nrf2 and GPX4 (Li S. J. et al., 2021). In the treatment of AD, Fer-1 was shown to ameliorate neuronal death and memory impairment *in vitro* and *in vivo* (Bao et al., 2021).

Hepcidin can reduce iron transport across the BBB and prevent iron overload in the brain. In cultured microvascular endothelial cells, hepcidin significantly inhibits the expression of FPN1, TfR1 and DMT1, and reduce iron uptake and release by neurons (Du et al., 2015). Hepcidin can reduce the iron level of mouse astrocortex and hippocampal neuron glial cells, reduce the formation of Aß plaques, and improve mouse cognitive function (Xu et al., 2020). The recombinant adenoviruses carrying the hepcidin gene could also reduce iron deposition and oxidative stress levels in the brain (Gong et al., 2016). Insamgobonhwan (GBH) can inhibit the impairment of ß-amyloid on cognitive function *in vivo*, and also inhibit cell death and lipid peroxidation *in vitro* cells. In addition, GBH restores ferritin GPX4, HO-1. The expression of COX-2 can improve cognitive dysfunction in mice, and it also has certain potential in AD treatment (Yang et al., 2022).

### VD

The main cause of VD is chronic cerebral hypoperfusion (CCH) caused by chronic cerebral blood flow (CBF) and a variety of vascular pathologies. These include atherosclerosis, arteriosclerosis, infarcts, white matter (WM) changes, and microbleeds (Calabrese et al., 2016; Kalaria, 2018). Researchers have demonstrated that amino acid metabolism is related to ferroptosis and that CCH can cause neuronal depolarization to release excess glutamate during the pathogenesis of VD, resulting in excitotoxicity, and high levels of glutamate inhibit the function of System Xc-. Glutamate excitotoxicity is also a pathological mechanism of iron toxicity, and iron chelation prevents excitotoxic cell death (Krzyzanowska et al., 2014; Liu et al., 2016; Frank et al., 2021). Nuclear factor erythroid 2 related factor 2 (Nrf2) is a fundamental regulator of cellular antioxidant defense systems, which regulates the expression of multiple antioxidant response element-dependent genes, including NADPH-quinone oxidoreductase 1 (NQO1), heme oxidoreductase 1 (HMOX1), ferritin heavy chain 1 (FTH1), FPN1, GSH, and GPX4 (Kerins and Ooi, 2018; Milkovic et al., 2019; Sarutipaiboon et al., 2020). Studies have shown that the expression level of Nrf2 is directly related to the susceptibility to iron poisoning. Increased expression of Nrf2 inhibits ferroptosis, and decreased expression promotes ferroptosis (Sun Y. R. et al., 2020; Fan et al., 2021; Nishizawa et al., 2022). Studies have shown that, on the one hand, Nrf2 promotes the expression of glutathione and GPX4 strengthens the function of the antioxidant system, and on the other hand, Nrf2 can also reduce intracellular iron accumulation by promoting the expression of ferritin and the simultaneous release of FPN1 storage and export. iron, thereby preventing iron poisoning (Yang et al., 2017; Kasai et al., 2019). The Nrf2 regulatory network plays a fundamental role in different mouse models of cerebral ischemia. Although the expression of Nrf2 is controversial in different studies, the neuroprotective effect of enhanced Nrf2/ARE activation has been demonstrated in different studies (Park et al., 2018; Liu et al., 2019, 2020). At the same time, NRF2 overexpression can improve cognitive dysfunction (Yang et al., 2014; Qi et al., 2018; Mao et al., 2019), suggesting that it may be related to the inhibition of iron poisoning, and the GSH metabolic network is a bridge connecting iron poisoning and VD.

CCH can also lead to massive iron deposition. Bilateral common carotid artery occlusion is the most commonly used experimental model for VD. In the study, it was found that CCH leads to iron deposition in the rat brain, and a large amount of iron deposition leads to neuronal death caused by oxidative stress. Among them, the CA1 area has the most iron deposition and neuronal death (Li et al., 2012; Du et al., 2018). Abnormal brain iron and iron ion deposition are closely related to cognitive dysfunction, and iron ion deposition has been confirmed in AD, PD, HD and other neurodegenerative diseases. It plays an important role in sexually transmitted diseases (Chen L. et al., 2019; Thomas et al., 2020; Xu et al., 2020). It has also been shown that a wide range of abnormal iron deposits in the cortex of patients with subcortical ischemic VD, especially in the lateral caudate nucleus, putamen, globus pallidus, and frontal cortex, correlate closely with the severity of cognitive impairment (Liu et al., 2015; Sun C. Y. et al., 2020). Model of cerebral ischemia-reperfusion injury confirmed that iron accumulation in the ischemic precursor is a novel mechanism of stroke injury, leading to neuronal death. Iron chelators attenuate ischemia-reperfusion injury in animal models (Tuo et al., 2017), indicating that iron-induced ferroptosis may be the underlying mechanism of VD neuron loss.

The oxidative stress produced by CCH has been proven to be one of the main pathogenic mechanisms leading to VD (Du et al., 2017; Lee et al., 2021), and studies have shown that blood lipid levels in VD patients are significantly higher than those in AD patients, suggesting lipid peroxidation. Having an important impact on the pathophysiology of VD, MDA levels may be a hallmark of VD (Gustaw-Rothenberg et al., 2010). Lipid peroxidation and ROS accumulation are key processes that induce iron toxicity (Dixon and Stockwell, 2014). LOX causes lipid peroxidation by catalyzing polyunsaturated fatty acids in phospholipid membranes, and inhibition of LOX inhibits ferroptosis (Kagan et al., 2017; Doll et al., 2019). During cerebral ischemia, the extensive increase in 12/15-LOX in brain tissue is an important cause of neuronal cell death and neurological damage. Inhibition of 12/15-LOX can reduce neuronal cell death and brain edema, and improve neurological prognosis (Piao et al., 2008; Pallast et al., 2010; Yigitkanli et al., 2017). In addition, NOX also plays an important role in lipid peroxidation. NOX1 expression was increased in hippocampal neurons during CCH, leading to lipid peroxidation and oxidative stress. It is an important cause of hippocampal neuronal degeneration and cognitive impairment (Choi et al., 2014). Lipid peroxidation caused by NOX is also one of the links of ferroptosis. NOX1 inhibitors have different effects on erastin-induced ferroptosis in Calu-1 cells and HT-1080 cells, and have a partial effect on HT-1080 cells (Dixon et al., 2012; Wang et al., 2021).

Thiazolidinediones such as rosiglitazone (ROSI), a drug for the treatment of diabetes, can selectively inhibit ACSL4 activity and thereby inhibit ferroptosis (Angeli et al., 2017; Doll et al., 2017). ACSL4 is widely expressed in brain tissue, especially in the hippocampal CA1 region, and the expression of ACSL4 is gradually increased during cerebral ischemia (Kassan et al., 2013; Peng et al., 2021). Rosiglitazone (ROSI) has been shown to reduce lipid peroxidation and oxidative stress injury in hippocampal neurons during CCH, protecting brain function (Sayan-Ozacmak et al., 2012). Multiple studies have shown that long-term use of pioglitazone in patients with insulin-dependent diabetes reduces the risk of dementia in nonpsychiatric patients (Heneka et al., 2015; Lu et al., 2018).

### PD

PD is the second most common neurodegenerative disease. It is common in middle-aged and elderly people with neurodegenerative diseases. Some PD patients also have cognitive dysfunction. In the late stage of PD, patients often have severe cognitive dysfunction such as dementia. Abnormal intracranial iron deposition is thought to be one of the pathogenic mechanisms of PD (Langkammer et al., 2016; An et al., 2018; Chen Q. et al., 2019), and iron induces the formation of Lewy bodies through oxidative stress pathway, which aggregates α-synuclein (Takahashi et al., 2007). Li et al. (2018) found that the iron content of the substantia nigra pars compacta in PD patients may gradually increase during the progression of PD and manifest as more significant iron deposition in the middle and late stages of the disease. Relatedly, observation of changes in its iron deposition may serve as a potential marker for monitoring disease progression. Substantia nigra iron deposition is also related to the cognitive function of patients (Liu et al., 2017). The QSM CP value of the left substantia nigra on MRI was negatively correlated with the ADL score. The CP value of the left frontal white matter was negatively correlated with the HAMD score, CP value of left substantia nigra and CP value of left frontal lobe were positively correlated with MOCA score, and CP value of left frontal lobe white matter was positively correlated with MMSE score. SWI reflects abnormal iron deposition in the brain through CP value, which can be used for the diagnosis of PD, but has little significance for disease staging, and can be used to study the mechanism of PD cognitive dysfunction and depression (Xiong et al., 2020). Quantitative analysis of susceptibility-weighted imaging (SWI) found that PD patients with mild cognitive impairment had increased iron concentrations in the globus pallidus and head of the caudate nucleus (Kim et al., 2021). Iron is also widely deposited in the premotor cortex, prefrontal lobe, insula, cerebellum, pons and other parts of PD patients (Acosta-Cabronero et al., 2017; Chen L. et al., 2019), which are all related to apathy and rapid eye movement sleep behavior disorder (RBD) and other non-motor symptoms. Tere was a metabolic disorder of iron in the cerebrospinal fluid of patients with PD combined with apathy and PD combined with RBD (Hu et al., 2015; Wang et al., 2016). Serum iron has also been found to be associated with anxiety in PD patients (Xu et al., 2018). In addition, Masaldan et al. (2019b) found that the abnormal accumulation of iron in PD may be closely related to the changes of iron regulatory proteins.

Alpha-synuclein (a-syn), as a key player in tyrosine hydroxylase-dependent dopamine synthesis and other dopamine metabolic processes (Do Van et al., 2016; Belarbi et al., 2017), may play a role in iron regulation (Duce et al., 2010; Zhou and Tan, 2017). In the presence of a copper catalyst, a-syn has ferroreductase potential, which, when combined with Fe3+ and converted to Fe2+, binds to the C-terminus of a-syn (Davies et al., 2011). Iron also increases the formation of non-normal fibers, a major event in PD (Abeyawardhane et al., 2018). Furthermore, GSH was found to be decreased in a mouse model of MPTP (Feng et al., 2014), while GSH depletion enhanced MPP+ toxicity in substantia nigra dopaminergic neurons (Wullner et al., 1996).

DFP has also been found to be neuroprotective in patients with early PD (Do Van et al., 2016). The MPP+-induced SH-SY5Y (a commonly used PD model) cell line is not programmed cell death and shares some similarities with iron toxicity: both involve lipid peroxidation and can be inhibited by DIM and Fer−1 suppressed. Results showed that iron chelators not only inhibited iron toxicity, but also protected dopamine neurons from cell death (Abeyawardhane et al., 2018). In a transgenic mouse model of PD, clioquinol was able to prevent the loss of substantia nigra cells due to the ability of clioquinol to chelate iron (Billings et al., 2016). Zeng et al. (2021) found that GPX4 in PD cells significantly decreased and ROS increased significantly after administration of ferric ammonium citrate, which in turn induced ferroptosis and led to neuronal death, while administration of iron chelators could inhibit ferroptosis and protect neurons.

### HD

HD is a progressive neurodegenerative disease characterized by rapid involuntary movements and cognitive impairment, ultimately leading to death, due to expansion of CAG repeats in the Huntingtin (HTT). The pathological hallmark of HD is iron accumulation and abnormal levels of glutamate and glutathione (Skouta et al., 2014; Agrawal et al., 2018). It has also been reported that plasma samples from HD patients have lower levels of GSH (Klepac et al., 2007) and lower GPX activity in erythrocytes, so the pathogenesis of HD may be related to ferroptosis. In a mouse model of HD, nitropropionic acid-treated mice also exhibited reduced global (cytoplasmic and mitochondrial) GSH reduction, suppressed hippocampal and cortical glutathione s-transferase (GST) function, and exhibited an HD phenotype (Klivenyi et al., 2000). Although the underlying mechanism by which mutant huntingtin causes neurodegeneration is unclear, the ability of huntingtin to induce oxidative damage has been demonstrated. A study found that Fer-1 treatment at 10 nM, 100 nM, and 1 μM protected neurons labeled with yellow fluorescent protein (YFP) and expressed by biotransfection with a pathogenic repeat (73Q) The huntingtin exon 1 fragment (mN90Q73) induces cell death. The number of medium spiny neurons (msnn) was significantly increased compared to controls (Skouta et al., 2014). The iron chelator DFO was protective and improved cognition in R6/2 mice, a mouse model of HD (Yang et al., 2016). Fer-1 can inhibit oxidative lipid damage and cell death in a cellular model of HD (Skouta et al., 2014).

### ALS

ALS is a neurodegenerative disease affecting motor neurons in the cortex, spinal cord, and brainstem, and clinically manifests as progressive muscle atrophy and weakness in the extremities and trunk. The pathogenesis of ALS is unknown, and neither is the treatment of the disease. The most common type of dementia associated with ALS is frontotemporal dementia (FTD), a progressive non-AD dementia syndrome characterized by localized frontal and temporal lobe degeneration (Ringbolz and Greene, 2006; Heidler-Gary and Hillis, 2007; Bede et al., 2018; Iridoy et al., 2019). Iron deposition was observed in the spinal cord of a mouse model of ALS (Golko-Perez et al., 2017). Abnormal iron deposits are found in ALS patients, and iron deposits were found in the motor cortex of the patient's brain at autopsy (Kwan et al., 2012). The lipid peroxidation of erythrocytes in ALS patients is significantly increased, while the content of GSH is decreased (Babu et al., 2008). The concentration of glutamate is higher, and the accumulation of glutamate can cause neuronal cell toxicity, which indicates that ferroptosis is directly involved in the pathogenesis of ALS mechanism. At the same time, motor neurons in ALS mice are very sensitive to GPX4 knockout-induced cell death (Conrad et al., 2018). At present, the only approved treatment for ALS in the United States and the European Union is the anti-excitatory amino acid toxicity drug Riluzole, which is a glutamate antagonist, which can inhibit the release of presynaptic glutamate and inhibit nerve endings. It can inhibit the neurotoxicity of excitatory amino acids by inhibiting the neurotoxicity of excitatory amino acids. It has a certain effect on ALS patients with bulbar palsy or limb paralysis as the first symptom, can delay the progression of ALS, and can clearly prolong the survival time and postponement of tracheostomy (Gurney et al., 1998).

### TBI

TBI is recognized as a disease with high mortality and complex survival, which is the main environmental risk factors for the development of neurodegenerative diseases. Complications of TBI are mainly motor function, cognitive function, and social dysfunction, which cause a serious burden on patients, family members and society, and age is an important factor affecting the prognosis of TBI (Griesbach et al., 2018; Fraser et al., 2019; Ritzel et al., 2019). Iron is considered to be an important agent of secondary injury after traumatic brain injury and can induce peroxidation and inflammation (Ayton and Lei, 2014). Studies have shown that after experimental brain trauma in rats, the production of lipid peroxidation products is significantly enhanced, the consumption of GSH and ascorbic acid is significantly increased (Bayir et al., 2002), and the activity of GPX is decreased (Xu et al., 2014). Elevated levels of 15-HpETE-PE after traumatic brain injury led to ferroptosis in the cerebral cortex and hippocampus, accompanied by increased expression of 15LO2 (a catalyst for the formation of protoferroporphyrin 15-oh-eicosapentaenoic acid) and depletion of GPX4, leading to cognitive impairment, effectively suggesting the possibility of ferroptosis (Wenzel et al., 2017).

Iron chelators such as DFO may improve cognitive function after traumatic brain injury (Khalaf et al., 2019). N,N'-bis(2-hydroxybenzyl)ethylenediamine-N,N'-diacetic acid hydrochloride (HBED) is a unique iron chelator that not only crosses the BBB, but also reduces the improvement and recovery of motor impairment and cognitive function after TBI in rats (Khalaf et al., 2019). The ferroptosis inhibitor Liproxstatin 1 can reduce brain edema and BBB permeability caused by TBI, improve motor and learning and memory impairment caused by TBI in rats, and significantly improve anxiety and cognitive function caused by TBI (Xie et al., 2019).

## Discussion

Ferroptosis is a newly discovered form of cell death manifested by iron overload, accumulation of lipid peroxidation, and ROS. The current research preliminarily showed that ferroptosis plays an important role in neurodegenerative diseases. Clinically, ferroptosis can be induced by the following methods and exert a neuroprotective effect: exogenous lipid supplementation promotes lipid peroxidation in cells; inhibition of GPX4 and expression of GSH; construction of nano-drug delivery system to supplement hydrogen peroxide and iron ions to promote Fenton reaction of tumor cells, etc. Therefore, ferroptosis is a potential target for the treatment of neurodegenerative diseases. However, the exploration of ferroptosis still faces many problems to be solved. First, the study of ferroptosis in cognitive dysfunction-related diseases is still in its infancy, and its underlying molecular mechanisms remain unclear. Although iron overload and lipid peroxidation can cause ferroptosis, their involvement in ferroptosis-related regulatory targets such as DMT1, FPN1, or iron uptake proteins needs to be further investigated. Second, there is no specific ferroptosis marker to comprehensively and extensively study its process, and which are the key executive molecules of ferroptosis remain unclear. Finally, it is known that abnormal iron metabolism can cause ferroptosis, and whether other metal elements induce ferroptosis remains to be explored.

In addition, ferroptosis is different from other forms of cell death, but these different forms of cell death are not independent of each other. The various forms of cell death are likely to be interconnected and form a network to participate in the regulation of cell death. Studies have found (Sun et al., 2018; Chen et al., 2021) that ferroptosis is closely related to apoptosis, and ferroptosis, autophagy and apoptosis can synergistically promote cancer cell death. Therefore, further studies on the relationship and mechanism between ferroptosis and other known cell death pathways are still needed in the future, which may be helpful for the treatment of cognitive dysfunction-related diseases. In addition, ferroptosis inhibitors contain some traditional ROS scavengers, but compared with traditional ROS scavengers, ferroptosis inhibitors can block iron-catalyzed ROS generation, activate oxidative stress, and induce cell death. It can only clear the accumulated ROS in cells and has a weak inhibitory effect on ferroptosis, and cannot completely block the occurrence of ferroptosis in cells. Various types of ferroptosis inducers and inhibitors have been found, but ferroptosis regulators generally suffer from low bioavailability and adverse reactions. Therefore, screening of ferroptosis-related drugs and traditional Chinese medicines with few adverse effects on normal tissues and high target specificity is crucial for the development of ferroptosis.

In conclusion, with the gradual deepening of ferroptosis research, the research on targeted drugs and new drug targets for ferroptosis is of great significance for the prevention and treatment of diseases in the future. At the same time, more and more experimental studies have confirmed the role of ferroptosis in neurodegenerative diseases, which provides more possibilities for the discovery of potential therapeutic drugs and therapeutic targets for neurodegenerative diseases, and also provides help to further explain the pathogenesis of neurodegenerative diseases. However, the research on ferroptosis in neurodegenerative diseases is still in its initial stage, and there are many unexplained problems, and more experiments are needed to deepen its understanding.

## Author contributions

YJi and ZZ conceived the study. KZ, SL, CR, YS, LT, and YJia performed literature searching and summary. YJi, HZ, and YJia wrote the manuscript. YJia and ZZ edited the manuscript. All authors contributed to the article and approved the submitted version.

## Funding

The work was supported by the National Natural Science Foundation of China (No. 82104244), Wuxi Municipal Health Commission (Nos. Q202050, Q202101, Q202167, M202167, and ZH202110), Wuxi Taihu Talent Project (Nos. WXTTP2020008 and WXTTP2021), Wuxi Medical Development Discipline Project (No. FZXK2021012), and Jiangsu Research Hospital Association for Precision Medication (JY202105).

## Conflict of interest

The authors declare that the research was conducted in the absence of any commercial or financial relationships that could be construed as a potential conflict of interest.

## Publisher's note

All claims expressed in this article are solely those of the authors and do not necessarily represent those of their affiliated organizations, or those of the publisher, the editors and the reviewers. Any product that may be evaluated in this article, or claim that may be made by its manufacturer, is not guaranteed or endorsed by the publisher.

## References

[B1] Abbott N. J. Ronnback L. Hansson E. (2006). Astrocyte-endothelial interactions at the blood-brain barrier. Nat. Rev. Neurosci. 7, 41–53. 10.1038/nrn1824 16371949

[B2] Abbruzzese G. Cossu G. Balocco M. Marchese R. Murgia D. Melis M.. (2011). A pilot trial of deferiprone for neurodegeneration with brain iron accumulation. Haematol-Hematol J. 96, 1708–1711. 10.3324/haematol.2011.043018 21791473 PMC3208690

[B3] Abeyawardhane D. L. Fernandez R. D. Murgas C. J. Heitger D. R. Forney A. K. Crozier M. K.. (2018). Iron redox chemistry promotes antiparallel oligomerization of alpha-synuclein. J. Am. Chem. Soc. 140, 5028–5032. 10.1021/jacs.8b02013 29608844

[B4] Acosta-Cabronero J. Cardenas-Blanco A. Betts M. J. Butryn M. Valdes-Herrera J. P. Galazky I.. (2017). The whole-brain pattern of magnetic susceptibility perturbations in Parkinson's disease. Brain. 140, 118–131. 10.1093/brain/aww278 27836833

[B5] Agrawal S. Fox J. Thyagarajan B. Fox J. H. (2018). Brain mitochondrial iron accumulates in Huntington's disease, mediates mitochondrial dysfunction, and can be removed pharmacologically. Free Radical Bio Med. 120, 317–329. 10.1016/j.freeradbiomed.2018.04.002 29625173 PMC5940499

[B6] Alborzinia N. Ignashkova T. I. Dejure F. R. Gendarme M. Theobald J. Wolfi S.. (2018). Golgi stress mediates redox imbalance and ferroptosis in human cells. Commun. Biol. 1, 210. 10.1038/s42003-018-0212-6 30511023 PMC6262011

[B7] An H. D. Zeng X. Y. Niu T. F. Li G. Y. Yang J. Zheng L. L.. (2018). Quantifying iron deposition within the substantia nigra of Parkinson's disease by quantitative susceptibility mapping. J. Neurol. Sci. 386, 46–52. 10.1016/j.jns.2018.01.008 29406966

[B8] Angeli J. P. F. Schneider M. Proneth B. Tyurina Y. Y. Tyurin V. A. Hammond V. J.. (2014). Inactivation of the ferroptosis regulator Gpx4 triggers acute renal failure in mice. Nat. Cell Biol. 16, 1180–U120. 10.1038/ncb3064 25402683 PMC4894846

[B9] Angeli J. P. F. Shah R. Pratt D. A. Conrad M. (2017). Ferroptosis Inhibition: Mechanisms and Opportunities. Trends Pharmacol. Sci. 38, 489–498. 10.1016/j.tips.2017.02.005 28363764

[B10] Ansari M. A. Scheff S. W. (2010). Oxidative stress in the progression of Alzheimer disease in the frontal cortex. J. Neuropathol. Exp. Neurol. 69, 155–167. 10.1097/NEN.0b013e3181cb5af4 20084018 PMC2826839

[B11] Ashraf A. Jeandriens J. Parkes H. G. So P. W. (2020). Iron dyshomeostasis, lipid peroxidation and perturbed expression of cystine/glutamate antiporter in Alzheimer?s disease: Evidence of ferroptosis. Redox Biol. 32:101494. 10.1016/j.redox.2020.101494 32199332 PMC7083890

[B12] Ayton S. Faux N. G. Bush A. I. (2017a). Association of cerebrospinal fluid ferritin level with preclinical cognitive decline in APOE-epsilon 4 carriers. JAMA Neurol. 74, 122–125. 10.1001/jamaneurol.2016.4406 27893873

[B13] Ayton S. Faux N. G. Bush A. I. Initia A. D. N. (2015). Ferritin levels in the cerebrospinal fluid predict Alzheimer's disease outcomes and are regulated by APOE. Nat Commun. 6, 6760. 10.1038/ncomms7760 25988319 PMC4479012

[B14] Ayton S. Fazlollahi A. Bourgeat P. Raniga P. Ng A. Lim Y. Y.. (2017b). Cerebral quantitative susceptibility mapping predicts amyloid-beta-related cognitive decline. Brain. 140, 2112–2119. 10.1093/brain/awx137 28899019

[B15] Ayton S. Lei P. (2014). Nigral iron elevation is an invariable feature of parkinson's disease and is a sufficient cause of neurodegeneration. Biomed Res. Int. 2014, 581256. 10.1155/2014/581256 24527451 PMC3914334

[B16] Ayton S. Wang Y. M. Diouf I. Schneider J. A. Brockman J. Morris M. C.. (2020). Brain iron is associated with accelerated cognitive decline in people with Alzheimer pathology. Mol Psychiatr. 25, 2932–2941. 10.1038/s41380-019-0375-7 30778133 PMC6698435

[B17] Babu G. N. Kumar A. Chandra R. Puri S. K. Singh R. L. Kalita J.. (2008). Oxidant-antioxidant imbalance in the erythrocytes of sporadic amyotrophic lateral sclerosis patients correlates with the progression of disease. Neurochem. Int. 52, 1284–1289. 10.1016/j.neuint.2008.01.009 18308427

[B18] Bao W. D. Pang P. Zhou X. T. Hu F. Xiong W. Chen K.. (2021). Loss of ferroportin induces memory impairment by promoting ferroptosis in Alzheimer's disease. Cell Death Differ. 28, 1548–1562. 10.1038/s41418-020-00685-9 33398092 PMC8166828

[B19] Bayir H. Kagan V. E. Tyurina Y. Y. Tyurin V. Ruppel R. A. Adelson P. D.. (2002). Assessment of antioxidant reserves and oxidative stress in cerebrospinal fluid after severe traumatic brain injury in infants and children. Pediatr. Res. 51, 571–578. 10.1203/00006450-200205000-00005 11978879

[B20] Becerril-Ortega J. Bordji K. Freret T. Rush T. Buisson A. (2014). Iron overload accelerates neuronal amyloid-beta production and cognitive impairment in transgenic mice model of Alzheimer's disease. Neurobiol. Aging. 35, 2288–2301. 10.1016/j.neurobiolaging.2014.04.019 24863668

[B21] Bede P. Omer T. Finegan E. Chipika R. H. Iyer P. M. Doherty M. A.. (2018). Connectivity-based characterisation of subcortical grey matter pathology in frontotemporal dementia and ALS: a multimodal neuroimaging study. Brain Imaging Behav. 12, 1696–1707. 10.1007/s11682-018-9837-9 29423814

[B22] Belaidi A. A. Bush A. I. (2016). Iron neurochemistry in Alzheimer's disease and Parkinson's disease: targets for therapeutics. J. Neurochem. 139, 179–197. 10.1111/jnc.13425 26545340

[B23] Belaidi A. A. Gunn A. P. Wong B. X. Ayton S. Appukuttan A. T. Roberts B. R.. (2018). Marked age-related changes in brain iron homeostasis in amyloid protein precursor knockout mice. Neurotherapeutics. 15, 1055–1062. 10.1007/s13311-018-0656-x 30112699 PMC6277293

[B24] Belarbi K. Cuvelier E. Destee A. Gressier B. Chartier-Harlin M. C. (2017). NADPH oxidases in Parkinson's disease: a systematic review. Mol. Neurodegener. 12. 10.1186/s13024-017-0225-5 29132391 PMC5683583

[B25] Ben Shachar D. Kahana N. Kampel V. Warshawsky A. Youdim M. B. H. (2004). Neuroprotection by a novel brain permeable iron chelator, VK-28, against 6-hydroxydopamine lession in rats. Neuropharmacology. 46, 254–263. 10.1016/j.neuropharm.2003.09.005 14680763

[B26] Billings J. L. Hare D. J. Nurjono M. Volitakis I. Cherny R. A. Bush A. I.. (2016). Effects of neonatal iron feeding and chronic clioquinol administration on the Parkinsonian human A53T transgenic mouse. ACS Chem. Neurosci. 7, 360–366. 10.1021/acschemneuro.5b00305 26712118

[B27] Bulk M. Abdelmoula W. M. Nabuurs R. J. A. van der Graaf L. M. Mulders C. W. H. Mulder A. A.. (2018a). Postmortem MRI and histology demonstrate differential iron accumulation and cortical myelin organization in early- and late-onset Alzheimer's disease. Neurobiol. Aging. 62, 231–242. 10.1016/j.neurobiolaging.2017.10.017 29195086

[B28] Bulk M. Kenkhuis B. van der Graaf L. M. Goeman J. J. Natte R. van der Weerd L.. (2018b). Postmortem T-2^*^ - weighted MRI imaging of cortical iron reflects severity of Alzheimer's disease. J. Alzheimers. Dis. 65, 1125–1137. 10.3233/JAD-180317 30103327 PMC6218127

[B29] Burdo J. R. Menzies S. L. Simpson I. A. Garrick L. M. Garrick M. D. Dolan K. G.. (2001). Distribution of divalent metal transporter 1 and metal transport protein 1 in the normal and Belgrade rat. J. Neurosci. Res. 66, 1198–1207. 10.1002/jnr.1256 11746453

[B30] Calabrese V. Giordano J. Signorile A. Ontario M. L. Castorina S. Pasquale D.. (2016). Major pathogenic mechanisms in vascular dementia: roles of cellular stress response and hormesis in neuroprotection. J. Neurosci. Res. 94, 1588–1603. 10.1002/jnr.23925 27662637

[B31] Cardoso B. R. Roberts B. R. Malpas C. B. Vivash L. Genc S. Saling M. M.. (2019). Supranutritional sodium selenate supplementation delivers selenium to the central nervous system: results from a randomized controlled pilot trial in Alzheimer's disease. Neurotherapeutics. 16, 192–202. 10.1007/s13311-018-0662-z 30215171 PMC6361071

[B32] Chan A. Shea T. B. (2006). Dietary and genetically-induced oxidative stress alter tau phosphorylation: Influence of folate and apolipoprotein E deficiency. J. Alzheimers. Dis. 9, 399–405. 10.3233/JAD-2006-9405 16917148

[B33] Chen L. Hua J. Ross C. A. Cai S. van Zijl P. C. M. Altered L.. (2019). brain iron content and deposition rate in Huntington's disease as indicated by quantitative susceptibility MRI. J. Neurosci. Res. 97, 467–479. 10.1002/jnr.24358 30489648 PMC6367012

[B34] Chen Q. Chen Y. Zhang Y. Wang F. Yu H. Zhang C.. (2019). Iron deposition in Parkinson's disease by quantitative susceptibility mapping. BMC Neurosci. 20:23. 10.1186/s12868-019-0505-9 31117957 PMC6532252

[B35] Chen S. W. Bu D. F. Zhu J. Yue T. H. Guo S. A. Wang X.. (2021). Endogenous hydrogen sulfide regulates xCT stability through persulfidation of OTUB1 at cysteine 91 in colon cancer cells. Neoplasia. 23, 461–472. 10.1016/j.neo.2021.03.009 33878705 PMC8081877

[B36] Choi D. H. Kim J. H. Seo J. H. Lee J. Choi W. S. Kim Y. S.. (2014). Matrix metalloproteinase-3 causes dopaminergic neuronal death through nox1-regenerated oxidative stress. PLoS ONE. 9:e115954. 10.1371/journal.pone.0115954 25536219 PMC4275264

[B37] Connor J. R. Menzies S. L. Stmartin S. M. Mufson E. J. A. (1992). Histochemical-study of iron, transferrin, and ferritin in Alzheimers diseased brains. J. Neurosci. Res. 31, 75–83. 10.1002/jnr.490310111 1613823

[B38] Conrad M. Kagan V. E. Bayir H. Pagnussat G. C. Head B. Traber M. G.. (2018). Regulation of lipid peroxidation and ferroptosis in diverse species. Gene Dev. 32, 602–619. 10.1101/gad.314674.118 29802123 PMC6004068

[B39] Crouch P. J. Savva M. S. Hung L. W. Donnelly P. S. Mot A. I. Parker S. J.. (2011). The Alzheimer's therapeutic PBT2 promotes amyloid-beta degradation and GSK3 phosphorylation via a metal chaperone activity. J. Neurochem. 119, 220–230. 10.1111/j.1471-4159.2011.07402.x 21797865

[B40] da Rocha T. J. Alves M. S. Guisso C. C. de Andrade F. M. Camozzato A. Oliveira A.. (2018). Association of GPX1 and GPX4 polymorphisms with episodic memory and Alzheimer's disease. Neurosci. Lett. 666, 32–37. 10.1016/j.neulet.2017.12.026 29246792

[B41] Davies P. Moualla D. Brown D. R. (2011). Alpha-synuclein is a cellular ferrireductase. PLoS ONE. 6, e15814. 10.1371/journal.pone.0015814 21249223 PMC3018422

[B42] Dixon S. J. Lemberg K. M. Lamprecht M. R. Skouta R. Zaitsev E. M. Gleason C. E.. (2012). Ferroptosis: an iron-dependent form of nonapoptotic cell death. Cell. 149, 1060–1072. 10.1016/j.cell.2012.03.042 22632970 PMC3367386

[B43] Dixon S. J. Stockwell B. R. (2014). The role of iron and reactive oxygen species in cell death. Nat. Chem. Biol. 10, 9–17. 10.1038/nchembio.1416 24346035

[B44] Do Van B. Gouel F. Jonneaux A. Timmerman K. Gele P. Petrault M.. (2016). Ferroptosis, a newly characterized form of cell death in Parkinson's disease that is regulated by PKC. Neurobiol. Dis. 94, 169–178. 10.1016/j.nbd.2016.05.011 27189756

[B45] Doll S. Freitas F. P. Shah R. Aldrovandi M. da Silva M. C. Ingold I.. (2019). FSP1 is a glutathione-independent ferroptosis suppressor. Nature. 575, 693. 10.1038/s41586-019-1707-0 31634899

[B46] Doll S. Proneth B. Tyurina Y. Y. Panzilius E. Kobayashi S. (2017). IngoId I, et al. ACSL4 dictates ferroptosis sensitivity by shaping cellular lipid composition. Nat. Chem. Biol. 13, 91–98. 10.1038/nchembio.2239 27842070 PMC5610546

[B47] Dolotov O. V. Inozemtseva L. S. Myasoedov N. F. Grivennikov I. A. (2022). Stress-induced depression and Alzheimer's disease: focus on astrocytes. Int. J. Mol. Sci. 23:4999. 10.3390/ijms23094999 35563389 PMC9104432

[B48] Donley D. W. Realing M. Gigley J. P. Fox J. H. (2021). Iron activates microglia and directly stimulates indoleamine-2,3-dioxygenase activity in the N171-82Q mouse model of Huntington's disease. PLoS ONE. 16:e0250606. 10.1371/journal.pone.0250606 33989290 PMC8121302

[B49] Du F. Qian Z. M. Luo Q. Q. Yung W. H. Ke Y. (2015). Hepcidin suppresses brain iron accumulation by downregulating iron transport proteins in iron-overloaded rats. Mol. Neurobiol. 52, 101–114. 10.1007/s12035-014-8847-x 25115800

[B50] Du L. Zhao Z. F. Cui A. L. Zhu Y. J. Zhang L. Liu J.. (2018). Increased iron deposition on brain quantitative susceptibility mapping correlates with decreased cognitive function in Alzheimer's disease. ACS Chem. Neurosci. 9, 1849–1857. 10.1021/acschemneuro.8b00194 29722955

[B51] Du S. Q. Wang X. R. Xiao L. Y. Tu J. F. Zhu W. He T.. (2017). Molecular mechanisms of vascular dementia: what can be learned from animal models of chronic cerebral hypoperfusion? Mol. Neurobiol. 54, 3670–3682. 10.1007/s12035-016-9915-1 27206432

[B52] Duce J. A. Tsatsanis A. Cater M. A. James S. A. Robb E. Wikhe K.. (2010). Iron-Export Ferroxidase Activity of beta-Amyloid Precursor Protein Is Inhibited by Zinc in Alzheimer's Disease. Cell. 142, 857–867. 10.1016/j.cell.2010.08.014 20817278 PMC2943017

[B53] Eid R. Arab N. T. T. Greenwood M. T. (2017). Iron mediated toxicity and programmed cell death: a review and a re-examination of existing paradigms. Bba-Mol. Cell Res. 1864, 399–430. 10.1016/j.bbamcr.2016.12.002 27939167

[B54] Fan G. H. Zhu T. Y. Min X. P. Juan X. (2021). Melatonin protects against PM2.5-induced lung injury by inhibiting ferroptosis of lung epithelial cells in a Nrf2-dependent manner. Ecotox Environ Safe. 223, 112588. 10.1016/j.ecoenv.2021.112588 34364124

[B55] Farina N. Llewellyn D. Isaac M. G. E. N. Tabet N. (2017). Vitamin E for Alzheimer's dementia and mild cognitive impairment. Cochrane Db Syst Rev. 1:CD002845. 10.1002/14651858.CD002854.pub4 28128435 PMC6464807

[B56] Fava A. Pirritano D. Plastino M. Cristiano D. Puccio G. Colica C.. (2013). The effect of lipoic acid therapy on cognitive functioning in patients with Alzheimer's disease. J Neurodegener Dis. 2013, 454253. 10.1155/2013/454253 26316990 PMC4437336

[B57] Feng G. S. Zhang Z. J. Bao Q. Q. Zhang Z. J. Zhou L. B. Jiang J.. (2014). Protective effect of chinonin in MPTP-induced C57BL/6 mouse model of Parkinson's disease. Biol. Pharm. Bull. 37, 1301–1307. 10.1248/bpb.b14-00128 24871044

[B58] Frank C. Hoffmann T. Zelder O. Felle M. F. Bremer E. (2021). Enhanced Glutamate Synthesis and Export by the Thermotolerant Emerging Industrial Workhorse Bacillus methanolicus in Response to High Osmolarity. Front. Microbiol. 12:640980. 10.3389/fmicb.2021.640980 33897645 PMC8060640

[B59] Fraser E. E. Downing M. G. Biernacki K. McKenzie D. P. Ponsford J. L. (2019). Cognitive reserve and age predict cognitive recovery after mild to severe traumatic brain injury. J Neurotraum. 36, 2753–2761. 10.1089/neu.2019.6430 31017049

[B60] Gallie B. L. Olivieri N. F. (2019). The role of deferiprone in iron chelation. New Engl. J. Med. 380, 891–93. 10.1056/NEJMc1817335 30811926

[B61] Gaschler M. M. Andia A. A. Liu H. R. Csuka J. M. Hurlocker B. Vaiana C. A.. (2018). FINO2 initiates ferroptosis through GPX4 inactivation and iron oxidation. Nat Chem Biol. 14, 507–15. 10.1038/s41589-018-0031-6 29610484 PMC5899674

[B62] Giometto B. Bozza F. Argentiero V. Gallo P. Pagni S. Piccinno M. G.. (1990). Transferrin receptors in rat central-nervous-system - an immunocytochemical study. J. Neurol. Sci. 98, 81–90. 10.1016/0022-510X(90)90183-N 2230832

[B63] Golko-Perez S. Amit T. Bar-Am O. Youdim M. B. H. Weinreb O. A. (2017). Novel iron chelator-radical scavenger ameliorates motor dysfunction and improves life span and mitochondrial biogenesis in SOD1(G93A) ALS mice. Neurotox. Res. 31, 230–244. 10.1007/s12640-016-9677-6 27826939

[B64] Gong J. Du F. Qian Z. M. Luo Q. Q. Sheng Y. Yung W. H.. (2016). Pre-treatment of rats with ad-hepcidin prevents iron-induced oxidative stress in the brain. Free Radical Bio Med. 90, 126–132. 10.1016/j.freeradbiomed.2015.11.016 26582371

[B65] Griesbach G. S. Masel B. E. Helvie R. E. Ashley M. J. (2018). The impact of traumatic brain injury on later life: effects on normal aging and neurodegenerative diseases. J Neurotraum. 35, 17–24. 10.1089/neu.2017.5103 28920532

[B66] Grossi C. Francese S. Casini A. Rosi M. C. Luccarini I. Fiorentini A.. (2009). Clioquinol decreases amyloid-beta burden and reduces working memory impairment in a transgenic mouse model of Alzheimer's disease. J. Alzheimers. Dis. 17, 423–440. 10.3233/JAD-2009-1063 19363260

[B67] Guillemot J. Canuel M. Essalmani R. Prat A. Seidah N. G. (2013). Implication of the proprotein convertases in iron homeostasis: proprotein convertase 7 sheds human transferrin receptor 1 and furin activates hepcidin. Hepatology. 57, 2514–2524. 10.1002/hep.26297 23390091

[B68] Guo C. Wang T. Zheng W. Shan Z. Y. Teng W. P. Wang Z. Y.. (2013). Intranasal deferoxamine reverses iron-induced memory deficits and inhibits amyloidogenic APP processing in a transgenic mouse model of Alzheimer's disease. Neurobiol. Aging. 34, 562–575. 10.1016/j.neurobiolaging.2012.05.009 22717236

[B69] Gurney M. E. Fleck T. J. Himes C. S. Hall E. D. (1998). Riluzole preserves motor function in a transgenic model of familial amyotrophic lateral sclerosis. Neurology. 50, 62–66. 10.1212/WNL.50.1.62 9443458

[B70] Gustaw-Rothenberg K. Kowalczuk K. Stryjecka-Zimmer M. (2010). Lipids' peroxidation markers in Alzheimer's disease and vascular dementia. Geriatr. Gerontol. Int. 10, 161–166. 10.1111/j.1447-0594.2009.00571.x 20446930

[B71] Gwon A. R. Park J. S. Park J. H. Baik S. H. Jeong H. Y. Hyun D. H.. (2010). Selenium attenuates A beta production and A beta-induced neuronal death. Neurosci. Lett. 469, 391–395. 10.1016/j.neulet.2009.12.035 20026385

[B72] Ha C. Ryu J. Park C. B. (2007). Metal ions differentially influence the aggregation and deposition of Alzheimer's beta-amyloid on a solid template. Biochemistry-Us. 46, 6118–6125. 10.1021/bi7000032 17455909

[B73] Habib E. Linher-Melville K. Lin H. X. Singh G. (2015). Expression of xCT and activity of system xc(-) are regulated by NRF2 in human breast cancer cells in response to oxidative stress. Redox Biol. 5, 33–42. 10.1016/j.redox.2015.03.003 25827424 PMC4392061

[B74] Hambright W. S. Fonseca R. S. Chen L. J. Na R. Ran Q. T. (2017). Ablation of ferroptosis regulator glutathione peroxidase 4 in forebrain neurons promotes cognitive impairment and neurodegeneration. Redox Biol. 12, 8–17. 10.1016/j.redox.2017.01.021 28212525 PMC5312549

[B75] He Y. J. Liu X. Y. Xing L. Wan X. Chang X. Jiang H. L.. (2020). Fenton reaction-independent ferroptosis therapy via glutathione and iron redox couple sequentially triggered lipid peroxide generator. Biomaterials. 241, 119911. 10.1016/j.biomaterials.2020.119911 32143060

[B76] Heidler-Gary J. Hillis A. E. (2007). Distinctions between the dementia in amyotrophic lateral sclerosis with frontotemporal dementia and the dementia of Alzheimer's disease. Amyotroph Lateral Sc. 8, 276–282. 10.1080/17482960701381911 17917849

[B77] Heneka M. T. Fink A. Doblhammer G. (2015). Effect of pioglitazone medication on the incidence of dementia. Ann. Neurol. 78, 284–294. 10.1002/ana.24439 25974006

[B78] Hentze M. W. Muckenthaler M. U. Galy B. Camaschella C. (2010). Two to tango: regulation of mammalian iron metabolism. Cell. 142, 24–38. 10.1016/j.cell.2010.06.028 20603012

[B79] Hider R. C. Kong X. L. (2011). Glutathione: a key component of the cytoplasmic labile iron pool. Biometals. 24, 1179–1187. 10.1007/s10534-011-9476-8 21769609

[B80] Holland R. McIntosh A. L. Finucane O. M. Mela V. Rubio-Araiz A. Timmons G.. (2018). Inflammatory microglia are glycolytic and iron retentive and typify the microglia in APP/PS1 mice. Brain Behav. Immun. 68, 183–196. 10.1016/j.bbi.2017.10.017 29061364

[B81] Hu Y. Yu S. Y. Zuo L. J. Piao Y. S. Cao C. J. Wang F.. (2015). Investigation on Abnormal Iron Metabolism and Related Inflammation in Parkinson Disease Patients with Probable RBD. PLoS ONE. 10, e0138997. 10.1371/journal.pone.0138997 26431210 PMC4592206

[B82] Ingold I. Berndt C. Schmitt S. Doll S. Poschmann G. Buday K.. (2018). Selenium Utilization by GPX4 Is required to prevent hydroperoxide-induced ferroptosis. Cell. 172, 409–22.e21. 10.1016/j.cell.2017.11.048 29290465

[B83] Iridoy M. O. Zubiri I. Zelaya M. V. Martinez L. Ausin K. Lachen-Montes M.. (2019). Neuroanatomical quantitative proteomics reveals common pathogenic biological routes between amyotrophic lateral sclerosis (ALS) and frontotemporal dementia (FTD). Int. J. Mol. Sci. 20, 4. 10.3390/ijms20010004 30577465 PMC6337647

[B84] Iwata-Ichikawa E. Kondo Y. Miyazaki I. Asanuma M. Ogawa N. (1999). Glial cells protect neurons against oxidative stress via transcriptional up-regulation of the glutathione synthesis. J. Neurochem. 72, 2334–2344. 10.1046/j.1471-4159.1999.0722334.x 10349842

[B85] James S. A. Churches Q. I. de Jonge M. D. Birchall I. E. Streltsov V. McColl G.. (2017). Iron, copper, and zinc concentration in A beta plaques in the APP/PS1 mouse model of Alzheimer's disease correlates with metal levels in the surrounding neuropil. ACS Chem. Neurosci. 8, 629–637. 10.1021/acschemneuro.6b00362 27958708

[B86] Kagan V. E. Mao G. W. Qu F. Angeli J. P. F. Doll S. St Croix C.. (2017). Oxidized arachidonic and adrenic PEs navigate cells to ferroptosis. Nat. Chem. Biol. 13, 81–90. 10.1038/nchembio.2238 27842066 PMC5506843

[B87] Kalaria R. N. (2018). The pathology and pathophysiology of vascular dementia. Neuropharmacology. 134, 226–239. 10.1016/j.neuropharm.2017.12.030 29273521

[B88] Karelson E. Bogdanovic N. Garlind A. Winblad B. Zilmer K. Kullisaar T.. (2001). The cerebrocortical areas in normal brain aging and in Alzheimer's disease: NOTICEABLE differences in the lipid peroxidation level and in antioxidant defense. Neurochem. Res. 26, 353–361. 10.1023/A:1010942929678 11495345

[B89] Kasai S. Yamazaki H. Tanji K. Engler M. J. Matsumiya T. Itoh K.. (2019). Role of the ISR-ATF4 pathway and its cross talk with Nrf2 in mitochondrial quality control. J. Clin. Biochem. Nutr. 64, 1–12. 10.3164/jcbn.18-37 30705506 PMC6348405

[B90] Kassan A. Herms A. Fernandez-Vidal A. Bosch M. Schieber N. L. Reddy B. J. N.. (2013). Acyl-CoA synthetase 3 promotes lipid droplet biogenesis in ER microdomains. J. Cell Biol. 203, 985–1001. 10.1083/jcb.201305142 24368806 PMC3871434

[B91] Kenkhuis B. Somarakis A. de Haan L. Dzyubachyk O. IJsselsteijn M. E. de Miranda NFCC. (2021). Iron loading is a prominent feature of activated microglia in Alzheimer's disease patients. Acta Neuropathol Com. 9:27. 10.1186/s40478-021-01126-5 33597025 PMC7887813

[B92] Kerins M. J. Ooi A. (2018). The roles of NRF2 in modulating cellular iron homeostasis. Antioxid Redox Sign. 29, 1756–1773. 10.1089/ars.2017.7176 28793787 PMC6208163

[B93] Khalaf S. Ahmad A. S. Chamara K. V. D. R. Dore S. (2019). Unique properties associated with the brain penetrant iron chelator HBED reveal remarkable beneficial effects after brain trauma. J. Neurotraum. 36, 43–53. 10.1089/neu.2017.5617 29743006 PMC6306957

[B94] Kim A. C. Lim S. Kim Y. K. (2018). Metal ion effects on A and Tau aggregation. Int. J. Mol. Sci. 19:128. 10.3390/ijms19010128 29301328 PMC5796077

[B95] Kim M. Yoo S. Kim D. Cho J. W. Kim J. S. Ahn J. H.. (2021). Extra-basal ganglia iron content and non-motor symptoms in drug-naive, early Parkinson's disease. Neurol. Sci. 42, 5297–5304. 10.1007/s10072-021-05223-0 33860863 PMC8642382

[B96] Klepac N. Relja M. Klepac R. Hecimovic S. Babic T. Trkulja V.. (2007). Oxidative stress parameters in plasma of Huntington's disease patients, asymptomatic Huntington's disease gene carriers and healthy subjects. J. Neurol. 254, 1676–1683. 10.1007/s00415-007-0611-y 17990062

[B97] Klivenyi P. Andreassen O. A. Ferrante R. J. Dedeoglu A. Mueller G. Lancelot E.. (2000). Mice deficient in cellular glutathione peroxidase show increased vulnerability to malonate, 3-nitropropionic acid, and 1-methyl-4-phenyl-1,2,5,6-tetrahydropyridine. J. Neurosci. 20, 1–7. 10.1523/JNEUROSCI.20-01-00001.2000 10627575 PMC6774090

[B98] Klopstock T. Tricta F. Neumayr L. Karin I. Zorzi G. Fradette C.. (2019). Safety and efficacy of deferiprone for pantothenate kinase-associated neurodegeneration: a randomised, double-blind, controlled trial and an open-label extension study. Lancet Neurol. 18, 631–642. 10.1016/S1474-4422(19)30142-5 31202468

[B99] Kress G. J. Dineley K. E. Reynolds I. J. (2002). The relationship between intracellular free iron and cell injury in cultured neurons, astrocytes, and oligodendrocytes. J. Neurosci. 22, 5848–5855. 10.1523/JNEUROSCI.22-14-05848.2002 12122047 PMC6757914

[B100] Krzyzanowska W. Pomierny B. Filip M. Pera J. (2014). Glutamate transporters in brain ischemia: to modulate or not? Acta Pharmacol. Sin. 35, 444–462. 10.1038/aps.2014.1 24681894 PMC4813728

[B101] Kwan J. Y. Jeong S. Y. Van Gelderen P. Deng H. X. Quezado M. M. Danielian L. E.. (2012). Iron Accumulation in deep cortical layers accounts for MRI signal abnormalities in ALS: correlating 7 Tesla MRI and pathology. PLoS ONE. 7, e35241. 10.1371/journal.pone.0035241 22529995 PMC3328441

[B102] Lane D. J. R. Metselaar B. Greenough M. Bush A. I. Ayton S. J. (2021). Ferroptosis and NRF2: an emerging battlefield in the neurodegeneration of Alzheimer's disease. Essays Biochem. 65, 925–940. 10.1042/EBC20210017 34623415

[B103] Langkammer C. Pirpamer L. Seiler S. Deistung A. Schweser F. Franthal S.. (2016). Quantitative susceptibility mapping in Parkinson's disease. PLoS ONE. 11:e0162460. 10.1371/journal.pone.0162460 27598250 PMC5012676

[B104] Lee J. M. Lee J. H. Song M. K. Kim Y. J. (2021). NXP031 improves cognitive impairment in a chronic cerebral hypoperfusion-induced vascular dementia rat model through Nrf2 signaling. Int. J. Mol. Sci. 22:6285. 10.3390/ijms22126285 34208092 PMC8230952

[B105] Lei P. Ayton S. Finkelstein D. I. Spoerri L. Ciccotosto G. D. Wright D. K.. (2012). Tau deficiency induces parkinsonism with dementia by impairing APP-mediated iron export. Nat. Med. 18, 291–295. 10.1038/nm.2613 22286308

[B106] Leng F. D. Edison P. (2021). Neuroinflammation and microglial activation in Alzheimer disease: where do we go from here? Nat. Rev. Neurol. 17, 157–172. 10.1038/s41582-020-00435-y 33318676

[B107] Li D. T. H. Hui E. S. Chan Q. Yao N. Chua S. E. McAlonan G. M.. (2018). Quantitative susceptibility mapping as an indicator of subcortical and limbic iron abnormality in Parkinson's disease with dementia. Neuroimage-Clin. 20, 365–373. 10.1016/j.nicl.2018.07.028 30128274 PMC6096006

[B108] Li J. Li O. W. Jiang Z. G. Ghanbari H. A. (2013). Oxidative stress and neurodegenerative disorders. Int. J. Mol. Sci. 14, 24438–24475. 10.3390/ijms141224438 24351827 PMC3876121

[B109] Li S. J. Zhou C. G. Zhu Y. H. Chao Z. W. Sheng Z. Y. Zhang Y. X.. (2021). Ferrostatin-1 alleviates angiotensin II (Ang II)- induced inflammation and ferroptosis in astrocytes. Int. Immunopharmacol. 90, 107179. 10.1016/j.intimp.2020.107179 33278745

[B110] Li S. W. Zheng L. S. Zhang J. Liu X. J. Wu Z. M. (2021). Inhibition of ferroptosis by up-regulating Nrf2 delayed the progression of diabetic nephropathy. Free Radical. Bio. Med. 162, 435–449. 10.1016/j.freeradbiomed.2020.10.323 33152439

[B111] Li W. Y. Li W. Wang Y. Leng Y. Xia Z. Y. (2021). Inhibition of DNMT-1 alleviates ferroptosis through NCOA4 mediated ferritinophagy during diabetes myocardial ischemia/reperfusion injury. Cell Death Discov. 7, 267. 10.1038/s41420-021-00656-0 34588431 PMC8481302

[B112] Li Y. X. He Y. S. Guan Q. Liu W. C. Han H. J. Nie Z. Y.. (2012). Disrupted iron metabolism and ensuing oxidative stress may mediate cognitive dysfunction induced by chronic cerebral hypoperfusion. Biol. Trace Elem. Res. 150, 242–248. 10.1007/s12011-012-9455-0 22639386

[B113] Lin G. P. Zhu F. Y. Kanaan N. M. Asano R. Shirafuji N. Sasaki H.. (2021). clioquinol decreases levels of phosphorylated, truncated, and oligomerized Tau protein. Int. J. Mol. Sci. 22:12063. 10.3390/ijms222112063 34769495 PMC8584684

[B114] Liu J. M. Sun J. Wang F. Y. Yu X. C. Ling Z. X. Li H. X.. (2015). Neuroprotective effects of clostridium butyricum against vascular dementia in mice via metabolic butyrate. Biomed Res. Int. 2015:412946. 10.1155/2015/412946 26523278 PMC4615854

[B115] Liu L. Vollmer M. K. Ahmad A. S. Fernandez V. M. Kim H. Dore S.. (2019). Pretreatment with Korean red ginseng or dimethyl fumarate attenuates reactive gliosis and confers sustained neuroprotection against cerebral hypoxic-ischemic damage by an Nrf2-dependent mechanism. Free Radical Bio Med. 131, 98–114. 10.1016/j.freeradbiomed.2018.11.017 30458277 PMC6362849

[B116] Liu L. Vollmer M. K. Kelly M. G. Fernandez V. M. Fernandez T. G. Kim H.. (2020). Reactive gliosis contributes to Nrf2-dependent neuroprotection by pretreatment with dimethyl fumarate or korean red ginseng against hypoxic-ischemia: focus on hippocampal injury. Mol. Neurobiol. 57, 105–117. 10.1007/s12035-019-01760-0 31494826 PMC6980429

[B117] Liu W. X. Wang J. Xie Z. M. Xu N. Zhang G. F. Jia M.. (2016). Regulation of glutamate transporter 1 via BDNF-TrkB signaling plays a role in the anti-apoptotic and antidepressant effects of ketamine in chronic unpredictable stress model of depression. Psychopharmacology. 233, 405–415. 10.1007/s00213-015-4128-2 26514555

[B118] Liu Z. Shen H. C. Lian T. H. Mao L. Tang S. X. Sun L.. (2017). Iron deposition in substantia nigra: abnormal iron metabolism, neuroinflammatory mechanism and clinical relevance. Sci Rep-Uk. 7:14973. 10.1038/s41598-017-14721-1 29097764 PMC5668412

[B119] Lloret A. Badia M. C. Mora N. J. Pallardo F. V. Alonso M. D. Vina J.. (2009). Vitamin E paradox in Alzheimer's disease: it does not prevent loss of cognition and may even be detrimental. J. Alzheimers. Dis. 17, 143–149. 10.3233/JAD-2009-1033 19494439

[B120] Lu C. H. Yang C. Y. Li C. Y. Hsieh C. Y. Ou H. T. (2018). Lower risk of dementia with pioglitazone, compared with other second-line treatments, in metformin-based dual therapy: a population-based longitudinal study. Diabetologia. 61, 562–573. 10.1007/s00125-017-4499-5 29138876

[B121] Ma L. F. Zhang X. Yu K. K. Xu X. Chen T. X. Shi Y.. (2021). Targeting SLC3A2 subunit of system X-C(-) is essential for m(6)A reader YTHDC2 to be an endogenous ferroptosis inducer in lung adenocarcinoma. Free Radical Bio Med. 168, 25–43. 10.1016/j.freeradbiomed.2021.03.023 33785413

[B122] Makarov P. Kropf S. Wiswedel I. Augustin W. Schild L. (2006). Consumption of redox energy by glutathione metabolism contributes to hypoxia/reoxygenation-induced injury in astrocytes. Mol. Cell. Biochem. 286, 95–101. 10.1007/s11010-005-9098-y 16583144

[B123] Mancias J. D. Wang X. X. Gygi S. P. Harper J. W. Kimmelman A. C. (2014). Quantitative proteomics identifies NCOA4 as the cargo receptor mediating ferritinophagy. Nature. 509, 105. 10.1038/nature13148 24695223 PMC4180099

[B124] Mao L. Yang T. Li X. (2019). Protective effects of sulforaphane in experimental vascular cognitive impairment: contribution of the Nrf2 pathway. J. Cerebr Blood F Met. 39, 371. 10.1177/0271678X18764083 29533123 PMC6365596

[B125] Masaldan S. Belaidi A. A. Ayton S. Bush A. I. (2019a). Cellular senescence and iron dyshomeostasis in Alzheimer's Disease. Pharmaceuticals-Base. 12:93. 10.3390/ph12020093 31248150 PMC6630536

[B126] Masaldan S. Bush A. I. Devos D. Rolland A. S. Moreau C. (2019b). Striking while the iron is hot: Iron metabolism and ferroptosis in neurodegeneration. Free Radical Bio Med. 133, 221–233. 10.1016/j.freeradbiomed.2018.09.033 30266679

[B127] Mclachlan D. R. C. Dalton A. J. Kruck T. P. A. Bell M. Y. Smith W. L. Kalow W.. (1991). Intramuscular desferrioxamine in patients with Alzheimers-disease. Lancet. 337, 1304–1308. 10.1016/0140-6736(91)92978-B 1674295

[B128] Meng F. X. Hou J. M. Sun T. S. (2017). *In vivo* evaluation of microglia activation by intracranial iron overload in central pain after spinal cord injury. J. Orthop. Surg. Res. 12:75. 10.1186/s13018-017-0578-z 28521818 PMC5437601

[B129] Milkovic L. Tomljanovic M. Gasparovic A. C. Kujundzic R. N. Simunic D. Konjevoda P.. (2019). Nutritional stress in head and neck cancer originating cell lines: the sensitivity of the NRF2-NQO1 axis. Cells-Basel. 8:1001. 10.3390/cells8091001 31470592 PMC6769674

[B130] Miotto G. Rossetto M. Di Paolo M. L. Orian L. Venerando R. Roveri A.. (2020). Insight into the mechanism of ferroptosis inhibition by ferrostatin-1. Redox Biol. 28, 101328. 10.1016/j.redox.2019.101328 31574461 PMC6812032

[B131] Mishima E. (2022). The E2F1-IREB2 axis regulates neuronal ferroptosis in cerebral ischemia. Hypertens. Res. 45, 1085–1086. 10.1038/s41440-021-00837-5 34952951

[B132] Munoz P. Zavala G. Castillo K. Aguirre P. Hidalgo C. Nunez M. T.. (2006). Effect of iron on the activation of the MAPK/ERK pathway in PC12 neuroblastoma cells. Biol. Res. 39, 189–190. 10.4067/S0716-97602006000100021 16629179

[B133] Newcombe E. A. Camats-Perna J. Silva M. L. Valmas N. Huat T. J. Medeiros R.. (2018). Inflammation: the link between comorbidities, genetics, and Alzheimer's disease. J Neuroinflamm. 15:276. 10.1186/s12974-018-1313-3 30249283 PMC6154824

[B134] Nikseresht S. Bush A. I. Ayton S. (2019). Treating Alzheimer's disease by targeting iron. Brit. J Pharmacol. 176, 3622–3635. 10.1111/bph.14567 30632143 PMC6715619

[B135] Nishizawa H. Yamanaka M. Igarashi K. (2022). Ferroptosis: regulation by competition between NRF2 and BACH1 and propagation of the death signal. Febs J. 10.1111/febs.16382. [Epub ahead of print]. 35107212

[B136] Nnah I. C. Lee C. H. Wessling-Resnick M. (2020). Iron potentiates microglial interleukin-1 beta secretion induced by amyloid-beta. J. Neurochem. 154, 177–189. 10.1111/jnc.14906 31693761 PMC7565447

[B137] Osama A. Zhang J. Yao J. Yao X. Fang J. (2020). Nrf2: a dark horse in Alzheimer's disease treatment. Ageing Res. Rev. 64, 101206. 10.1016/j.arr.2020.101206 33144124

[B138] Ou M. Jiang Y. Ji Y. Zhou Q. Du Z. Zhu H.. (2022). Role and mechanism of ferroptosis in neurological diseases. Mol Metab. 61, 101502. 10.1016/j.molmet.2022.101502 35447365 PMC9170779

[B139] Pallast S. Arai K. Pekcec A. Yigitkanli K. Yu Z. Y. Wang X. Y.. (2010). Increased nuclear apoptosis-inducing factor after transient focal ischemia: a 12/15-lipoxygenase-dependent organelle damage pathway. J Cerebr Blood F Met. 30, 1157–1167. 10.1038/jcbfm.2009.281 20068575 PMC2915762

[B140] Park S. Y. Choi Y. W. Park G. (2018). Nrf2-mediated neuroprotection against oxygen-glucose deprivation/reperfusion injury by emodin via AMPK-dependent inhibition of GSK-3 beta. J. Pharm. Pharmacol. 70, 525–535. 10.1111/jphp.12885 29424025

[B141] Peng C. L. Jiang N. Zhao J. F. Liu K. Jiang W. Cao P. G.. (2021). Metformin relieves H/R-induced cardiomyocyte injury through miR-19a/ACSL axis - possible therapeutic target for myocardial I/R injury. Toxicol Appl Pharm. 414:115408. 10.1016/j.taap.2021.115408 33476677

[B142] Peters D. G. Pollack A. N. Cheng K. C. Sun D. X. Saido T. Haaf M. P.. (2018). Dietary lipophilic iron alters amyloidogenesis and microglial morphology in Alzheimer's disease knock-in APP mice. Metallomics. 10, 426–443. 10.1039/C8MT00004B 29424844

[B143] Piao Y. S. Du Y. C. Oshima H. Jin J. C. Nomura M. Yoshimoto T.. (2008). Platelet-type 12-lipoxygenase accelerates tumor promotion of mouse epidermal cells through enhancement of cloning efficiency. Carcinogenesis. 29, 440–447. 10.1093/carcin/bgm274 18174253

[B144] Qi Q. Q. Xu J. Lv P. Y. Dong Y. H. Liu Z. J. Hu M.. (2018). DL-3-n-butylphthalide alleviates vascular cognitive impairment induced by chronic cerebral hypoperfusion by activating the Akt/Nrf2 signaling pathway in the hippocampus of rats. Neurosci. Lett. 672, 59–64. 10.1016/j.neulet.2017.11.051 29175633

[B145] Raha A. A. Biswas A. Henderson J. Chakraborty S. Holland A. Friedland R. P.. (2022). Interplay of ferritin accumulation and ferroportin loss in ageing brain: implication for protein aggregation in down syndrome dementia, Alzheimer's, and Parkinson's diseases. Int. J. Mol. Sci. 23:1060. 10.3390/ijms23031060 35162984 PMC8834792

[B146] Rassu G. Soddu E. Cossu M. Brundu A. Cerri G. Marchetti N.. (2015). Solid microparticles based on chitosan or methyl-beta-cyclodextrin: A first formulative approach to increase the nose-to-brain transport of deferoxamine mesylate. J. Control. Release. 201, 68–77. 10.1016/j.jconrel.2015.01.025 25620068 PMC4330128

[B147] Rathnasamy G. Ling E. A. Kaur C. (2013). Consequences of iron accumulation in microglia and its implications in neuropathological conditions. Cns Neurol Disord-Dr. 12, 785–798. 10.2174/18715273113126660169 24047528

[B148] Ringbolz G. M. Greene S. R. (2006). The relationship between amyotrophic lateral sclerosis and frontotemporal dementia. Curr. Neurol. Neurosci. 6, 387–392. 10.1007/s11910-996-0019-6 16928348

[B149] Ritchie C. W. Bush A. I. Mackinnon A. Macfarlane S. Mastwyk M. MacGregor L.. (2003). Metal-protein attenuation with iodochlorhydroxyquin (clioquinol) targeting A beta amyloid deposition and toxicity in Alzheimer disease - A pilot phase 2 clinical trial. Arch Neurol-Chicago. 60, 1685–1691. 10.1001/archneur.60.12.1685 14676042

[B150] Ritzel R. M. Doran S. J. Glaser E. P. Meadows V. E. Faden A. I. Stoica B. A.. (2019). Old age increases microglial senescence, exacerbates secondary neuroinflammation, and worsens neurological outcomes after acute traumatic brain injury in mice. Neurobiol. Aging. 77, 194–206. 10.1016/j.neurobiolaging.2019.02.010 30904769 PMC6486858

[B151] Rottkamp C. A. Raina A. K. Zhu X. W. Gaier E. Bush A. I. Atwood C. S.. (2001). Redox-active iron mediates amyloid-beta toxicity. Free Radical Bio Med. 30, 447–450. 10.1016/S0891-5849(00)00494-9 11182300

[B152] Rouault T. A. (2006). The role of iron regulatory proteins in mammalian iron homeostasis and disease. Nat. Chem. Biol. 2, 406–414. 10.1038/nchembio807 16850017

[B153] Sanchez M. Galy B. Muckenthaler M. U. Hentze M. W. (2007). Iron-regulatory proteins limit hypoxia-inducible factor-2alpha expression in iron deficiency. Nat. Struct. Mol. Biol. 14, 420–426. 10.1038/nsmb1222 17417656

[B154] Sarutipaiboon I. Settasatian N. Komanasin N. Kukongwiriyapan U. Sawanyawisuth K. Intharaphet P.. (2020). Association of genetic variations in NRF2, NQO1, HMOX1, and MT with severity of coronary artery disease and related risk factors. Cardiovasc. Toxicol. 20, 176–189. 10.1007/s12012-019-09544-7 31332605

[B155] Sayan-Ozacmak H. Ozacmak V. H. Barut F. Jakubowska-Dogru E. (2012). Rosiglitazone treatment reduces hippocampal neuronal damage possibly through alleviating oxidative stress in chronic cerebral hypoperfusion. Neurochem. Int. 61, 287–290. 10.1016/j.neuint.2012.05.011 22609374

[B156] Schneider S. A. Hardy J. Bhatia K. P. (2012). Syndromes of neurodegeneration with brain iron accumulation (NBIA): an update on clinical presentations, histological and genetic underpinnings, and treatment considerations. Movement Disord. 27, 42–53. 10.1002/mds.23971 22031173

[B157] Seibt T. M. Proneth B. Conrad M. (2019). Role of GPX4 in ferroptosis and its pharmacological implication. Free Radical Bio Med. 133, 144–152. 10.1016/j.freeradbiomed.2018.09.014 30219704

[B158] Shih A. Y. Johnson D. A. Wong G. Kraft A. D. Jiang L. Erb H.. (2003). Coordinate regulation of glutathione biosynthesis and release by Nrf2-expressing glia potently protects neurons from oxidative stress. J. Neurosci. 23, 3394–3406. 10.1523/JNEUROSCI.23-08-03394.2003 12716947 PMC6742304

[B159] Shintoku R. Takigawa Y. Yamada K. Kubota C. Yoshimoto Y. Takeuchi T.. (2017). Lipoxygenase-mediated generation of lipid peroxides enhances ferroptosis induced by erastin and RSL3. Cancer Sci. 108, 2187–2194. 10.1111/cas.13380 28837253 PMC5666033

[B160] Silvestri L. Camaschella C. (2008). A potential pathogenetic role of iron in Alzheimer's disease. J. Cell Mol Med. 12, 1548–50. 10.1111/j.1582-4934.2008.00356.x 18466351 PMC3918070

[B161] Singh N. (2014). The role of iron in prion disease and other neurodegenerative diseases. PLoS Pathog. 10, e1009395. 10.1371/journal.ppat.1004335 25232824 PMC4169465

[B162] Skouta R. Dixon S. J. Wang J. L. Dunn D. E. Orman M. Shimada K.. (2014). Ferrostatins inhibit oxidative lipid damage and cell death in diverse disease models. J. Am. Chem. Soc. 136, 4551–4556. 10.1021/ja411006a 24592866 PMC3985476

[B163] Spotorno N. Acosta-Cabronero J. Stomrud E. Lampinen B. Strandberg O. T. van Westen D.. (2020). Relationship between cortical iron and tau aggregation in Alzheimer's disease. Brain. 143, 1341–1349. 10.1093/brain/awaa089 32330946 PMC7241946

[B164] Stockwell B. R. Angeli J. P. F. Bayir H. Bush A. I. Conrad M. Dixon S. J.. (2017). Ferroptosis: A regulated cell death nexus linking metabolism, redox biology, and disease. Cell. 171, 273–285. 10.1016/j.cell.2017.09.021 28985560 PMC5685180

[B165] Streit W. J. Rotter J. Winter K. Muller W. Khoshbouei H. Bechmann I.. (2022). Droplet degeneration of hippocampal and cortical neurons signifies the beginning of neuritic plaque formation. J. Alzheimers. Dis. 85, 1701–1720. 10.3233/JAD-215334 34958037

[B166] Sun C. Y. Wu Y. Ling C. Xie Z. Y. Kong Q. L. Fang X. J.. (2020). Deep gray matter iron deposition and its relationship to clinical features in cerebral autosomal dominant arteriopathy with subcortical infarcts and leukoencephalopathy patients A 7.0-T magnetic resonance imaging study. Stroke. 51, 1750–1757. 10.1161/STROKEAHA.119.028812 32397933

[B167] Sun Y. Zheng Y. F. Wang C. X. Liu Y. Z. (2018). Glutathione depletion induces ferroptosis, autophagy, and premature cell senescence in retinal pigment epithelial cells. Cell Death Dis. 9, 753. 10.1038/s41419-018-0794-4 29988039 PMC6037763

[B168] Sun Y. R. He L. B. Wang T. Y. Hua W. Qin H. Wang J. J.. (2020). Activation of p62-Keap1-Nrf2 pathway protects 6-hydroxydopamine-induced ferroptosis in dopaminergic cells. Mol. Neurobiol. 57, 4628–4641. 10.1007/s12035-020-02049-3 32770451

[B169] Takahashi M. Ko L. W. Kulathingal J. Jiang P. Z. Sevlever D. Yen S. H. C.. (2007). Oxidative stress-induced phosphorylation, degradation and aggregation of alpha-synuclein are linked to upregulated CK2 and cathepsin D. Eur. J. Neurosci. 26, 863–874. 10.1111/j.1460-9568.2007.05736.x 17714183

[B170] Tao Y. L. Wang Y. Rogers J. T. Wang F. D. (2014). Perturbed iron distribution in Alzheimer's disease serum, cerebrospinal fluid, and selected brain regions: a systematic review and meta-analysis. J. Alzheimers. Dis. 42, 679–690. 10.3233/JAD-140396 24916541

[B171] Thomas G. E. C. Leyland L. A. Schrag A. E. Lees A. J. Acosta-Cabronero J. Weil R. S.. (2020). Brain iron deposition is linked with cognitive severity in Parkinson's disease. J Neurol Neurosur Ps. 91, 418–425. 10.1136/jnnp-2019-322042 32079673 PMC7147185

[B172] Torii S. (2018). Lipid peroxide accumulation enhances iron-dependent cell death ferroptosis in cancer cells. Cancer Sci. 109, 586.

[B173] Tripathi A. K. Haldar S. Qian J. Beserra A. Suda S. Singh A.. (2015). Prion protein functions as a ferrireductase partner for ZIP14 and DMT1. Free Radical. Bio. Med. 84, 322–330. 10.1016/j.freeradbiomed.2015.03.037 25862412 PMC4476631

[B174] Tsatsanis A. McCorkindale A. N. Wong B. X. Patrick E. Ryan T. M. Evans R. W.. (2021). The acute phase protein lactoferrin is a key feature of Alzheimer's disease and predictor of A beta burden through induction of APP amyloidogenic processing. Mol Psychiatr. 26, 5516–5531. 10.1038/s41380-021-01248-1 34400772 PMC8758478

[B175] Tuo Q. Z. Lei P. Jackman K. A. Li X. I. Xiong H. Li X. L.. (2017). Tau-mediated iron export prevents ferroptotic damage after ischemic stroke. Mol Psychiatr. 22, 1520–1530. 10.1038/mp.2017.171 28886009

[B176] van Duijn S. Bulk M. van Duinen S. G. Nabuurs R. J. A. van Buchem M. A. van der Weerd L.. (2017). Cortical iron reflects severity of Alzheimer's disease. J. Alzheimers. Dis. 60, 1533–1545. 10.3233/JAD-161143 29081415 PMC5676973

[B177] Wan W. B. Cao L. Kalionis B. Murthi P. Xia S. J. Guan Y. T.. (2019). Iron deposition leads to hyperphosphorylation of Tau and disruption of insulin signaling. Front. Neurol. 2019, 10. 10.3389/fneur.2019.00607 31275224 PMC6593079

[B178] Wang F. Yu S. Y. Zuo L. J. Cao C. J. Hu Y. Chen Z. J.. (2016). Excessive iron and alpha-synuclein oligomer in brain are relevant to pure apathy in Parkinson disease. J. Geriatr. Psychiatry Neurol. 29, 187–194. 10.1177/0891988716632918 26940028

[B179] Wang J. F. Yin X. M. He W. Xue W. Zhang J. Huang Y. R.. (2021). SUV39H1 deficiency suppresses clear cell renal cell carcinoma growth by inducing ferroptosis. Acta Pharm. Sin. B. 11, 406–419. 10.1016/j.apsb.2020.09.015 33643820 PMC7893126

[B180] Wang T. Wang C. Y. Shan Z. Y. Teng W. P. Wang Z. Y. (2012). Clioquinol reduces Zinc accumulation in neuritic plaques and inhibits the amyloidogenic pathway in a beta PP/PS1 transgenic mouse brain. J. Alzheimers. Dis. 29, 549–559. 10.3233/JAD-2011-111874 22269164

[B181] Ward R. Zucca F. A. Duyn J. H. Crichton R. R. Zecca L. (2014). The role of iron in brain ageing and neurodegenerative disorders. Lancet Neurol. 13, 1045–1060. 10.1016/S1474-4422(14)70117-6 25231526 PMC5672917

[B182] Ward R. J. Dexter D. Florence A. Aouad F. Hider R. Jenner P.. (1995). Brain iron in the ferrocene-loaded rat - its chelation and influence on dopamine metabolism. Biochem. Pharmacol. 49, 1821–1826. 10.1016/0006-2952(94)00521-M 7598744

[B183] Wenzel S. E. Tyurina Y. Y. Zhao J. M. Croix C. M. S. Dar H. H. Mao G. W.. (2017). PEBP1 wardens ferroptosis by enabling lipoxygenase generation of lipid death signals. Cell. 171, 628. 10.1016/j.cell.2017.09.044 29053969 PMC5683852

[B184] Wullner U. Loschmann P. A. Schulz J. B. Schmid A. Dringen R. Eblen F.. (1996). Glutathione depletion potentiates MPTP and MPP(+) toxicity in nigral dopaminergic neurones. Neuroreport. 7, 921–923. 10.1097/00001756-199603220-00018 8724674

[B185] Xie B. S. Wang Y. Q. Lin Y. Mao Q. Feng J. F. Gao G. Y.. (2019). Inhibition of ferroptosis attenuates tissue damage and improves long-term outcomes after traumatic brain injury in mice. CNS Neurosci. Ther. 25, 465–475. 10.1111/cns.13069 30264934 PMC6488926

[B186] Xiong W. Li L. F. Huang L. Liu Y. Xia Z. C. Zhou X. X.. (2020). Different iron deposition patterns in akinetic/rigid-dominant and tremor-dominant Parkinson's disease. Clin Neurol Neurosur. 198:106181. 10.1016/j.clineuro.2020.106181 33022525

[B187] Xu J. G. Wang H. D. Ding K. Zhang L. Wang C. X. Li T.. (2014). Luteolin provides neuroprotection in models of traumatic brain injury via the Nrf2-ARE pathway. Free Radical Bio Med. 71, 186–195. 10.1016/j.freeradbiomed.2014.03.009 24642087

[B188] Xu W. Zhi Y. Yuan Y. S. Zhang B. F. Shen Y. T. Zhang H.. (2018). Correlations between abnormal iron metabolism and non-motor symptoms in Parkinson's disease. J. Neural Transm. 125, 1027–1032. 10.1007/s00702-018-1889-x 29748849

[B189] Xu Y. Zhang Y. T. Zhang J. H. Han K. Zhang X. W. Bai X.. (2020). Astrocyte hepcidin ameliorates neuronal loss through attenuating brain iron deposition and oxidative stress in APP/PS1 mice. Free Radical Bio Med. 158, 84–95. 10.1016/j.freeradbiomed.2020.07.012 32707154

[B190] Yang J. H. Nguyen C. D. Lee G. Na C. S. (2022). Insamgobonhwan protects neuronal cells from lipid ROS and improves deficient cognitive function. Antioxidants-Basel. 11:295. 10.3390/antiox11020295 35204177 PMC8868228

[B191] Yang W. S. Kim K. J. Gaschler M. M. Patel M. Shchepinov M. S. Stockwell B. R.. (2016). Peroxidation of polyunsaturated fatty acids by lipoxygenases drives ferroptosis. P. Natl. Acad. Sci. USA. 113, E4966–E75. 10.1073/pnas.1603244113 27506793 PMC5003261

[B192] Yang X. Y. Park S. H. Chang H. C. Shapiro J. S. Vassilopoulos A. Sawicki K. T.. (2017). Sirtuin 2 regulates cellular iron homeostasis via deacetylation of transcription factor NRF2. J. Clin. Invest. 127, 1505–1516. 10.1172/JCI88574 28287409 PMC5373873

[B193] Yang Y. Zhang J. J. Liu H. Zhang L. (2014). Change of Nrf2 expression in rat hippocampus in a model of chronic cerebral hypoperfusion. Int. J. Neurosci. 124, 577–584. 10.3109/00207454.2013.863196 24219386

[B194] Yigitkanli K. Zheng Y. Pekcec A. Lo E. H. van Leyen K. (2017). Increased 12/15-lipoxygenase leads to widespread brain injury following global cerebral ischemia. Transl. Stroke Res. 8, 194–202. 10.1007/s12975-016-0509-z 27838820 PMC5350054

[B195] Yoo M. H. Gu X. Xu X. M. Kim J. Y. Carlson B. A. Patterson A. D.. (2010). Delineating the role of glutathione peroxidase 4 in protecting cells against lipid hydroperoxide damage and in Alzheimer's disease. Antioxid. Redox Signal. 12, 819–827. 10.1089/ars.2009.2891 19769463 PMC2861544

[B196] Yoo S. E. Chen L. J. Na R. Liu Y. H. Rios C. Van Remmen H.. (2012). Gpx4 ablation in adult mice results in a lethal phenotype accompanied by neuronal loss in brain. Free Radical Bio Med. 52, 1820–1827. 10.1016/j.freeradbiomed.2012.02.043 22401858 PMC3341497

[B197] Zeng X. Y. An H. D. Yu F. Wang K. Zheng L. L. Zhou W.. (2021). Benefits of iron chelators in the treatment of Parkinson's disease. Neurochem. Res. 46, 1239–1251. 10.1007/s11064-021-03262-9 33646533 PMC8053182

[B198] Zhan S. Lu L. Pan S. S. Wei X. Q. Miao R. R. Liu X. H.. (2022). Targeting NQO1/GPX4-mediated ferroptosis by plumbagin suppresses *in vitro* and *in vivo* glioma growth. Brit. J. Cancer. 127, 364–376, 10.1038/s41416-022-01800-y 35396498 PMC9296534

[B199] Zhang Y. H. Wang D. W. Xu S. F. Zhang S. Fan Y. G. Yang Y. Y.. (2018). alpha-Lipoic acid improves abnormal behavior by mitigation of oxidative stress, inflammation, ferroptosis, and tauopathy in P301S Tau transgenic mice. Redox Biol. 14, 535–548. 10.1016/j.redox.2017.11.001 29126071 PMC5684493

[B200] Zhao Y. Y. Yang Y. Q. Sheng H. H. Tang Q. Han L. Wang S. M.. (2022). GPX4 plays a crucial role in fuzheng kang'ai decoction-induced non-small cell lung cancer cell ferroptosis. Front. Pharmacol. 13, 851680. 10.3389/fphar.2022.851680 35496303 PMC9043103

[B201] Zhou Z. D. Tan E. K. (2017). Iron regulatory protein (IRP)-iron responsive element (IRE) signaling pathway in human neurodegenerative diseases. Mol. Neurodegener. 12:75. 10.1186/s13024-017-0218-4 29061112 PMC5654065

[B202] Zhu K. Y. Zhu X. Liu S. Q. Yu J. Wu S. W. Hei M. Y.. (2022). Glycyrrhizin attenuates hypoxic-ischemic brain damage by inhibiting ferroptosis and neuroinflammation in neonatal rats via the HMGB1/GPX4 pathway. Oxid. Med. Cell Longev. 2022, 8438528. 10.1155/2022/8438528 35432719 PMC9010207

